# Psychedelic Drugs in Mental Disorders: Current Clinical Scope and Deep Learning‐Based Advanced Perspectives

**DOI:** 10.1002/advs.202413786

**Published:** 2025-03-20

**Authors:** Sung‐Hyun Kim, Sumin Yang, Jeehye Jung, Jeonghyeon Choi, Mingon Kang, Jae‐Yeol Joo

**Affiliations:** ^1^ Department of Pharmacy College of Pharmacy Hanyang University Ansan Gyeonggi‐do 15588 Republic of Korea; ^2^ Department of Computer Science University of Nevada Las Vegas NV 89154 USA

**Keywords:** artificial intelligence, deep learning, drug development, mental disorders, psychedelic drugs, transcriptome

## Abstract

Mental disorders are a representative type of brain disorder, including anxiety, major depressive depression (MDD), and autism spectrum disorder (ASD), that are caused by multiple etiologies, including genetic heterogeneity, epigenetic dysregulation, and aberrant morphological and biochemical conditions. Psychedelic drugs such as psilocybin and lysergic acid diethylamide (LSD) have been renewed as fascinating treatment options and have gradually demonstrated potential therapeutic effects in mental disorders. However, the multifaceted conditions of psychiatric disorders resulting from individuality, complex genetic interplay, and intricate neural circuits impact the systemic pharmacology of psychedelics, which disturbs the integration of mechanisms that may result in dissimilar medicinal efficiency. The precise prescription of psychedelic drugs remains unclear, and advanced approaches are needed to optimize drug development. Here, recent studies demonstrating the diverse pharmacological effects of psychedelics in mental disorders are reviewed, and emerging perspectives on structural function, the microbiota‐gut‐brain axis, and the transcriptome are discussed. Moreover, the applicability of deep learning is highlighted for the development of drugs on the basis of big data. These approaches may provide insight into pharmacological mechanisms and interindividual factors to enhance drug discovery and development for advanced precision medicine.

## Graphical abstract

Graphic illustration showing the strategic approaches based on the currently investigated evidence of psychedelics that suggests the challenges and how to address the prescription of mental disorders. These applicable avenues may provide substantial insight into distinguishing between brain networks and psychedelics.

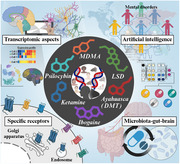

## 1 Introduction

**Figure 1:**
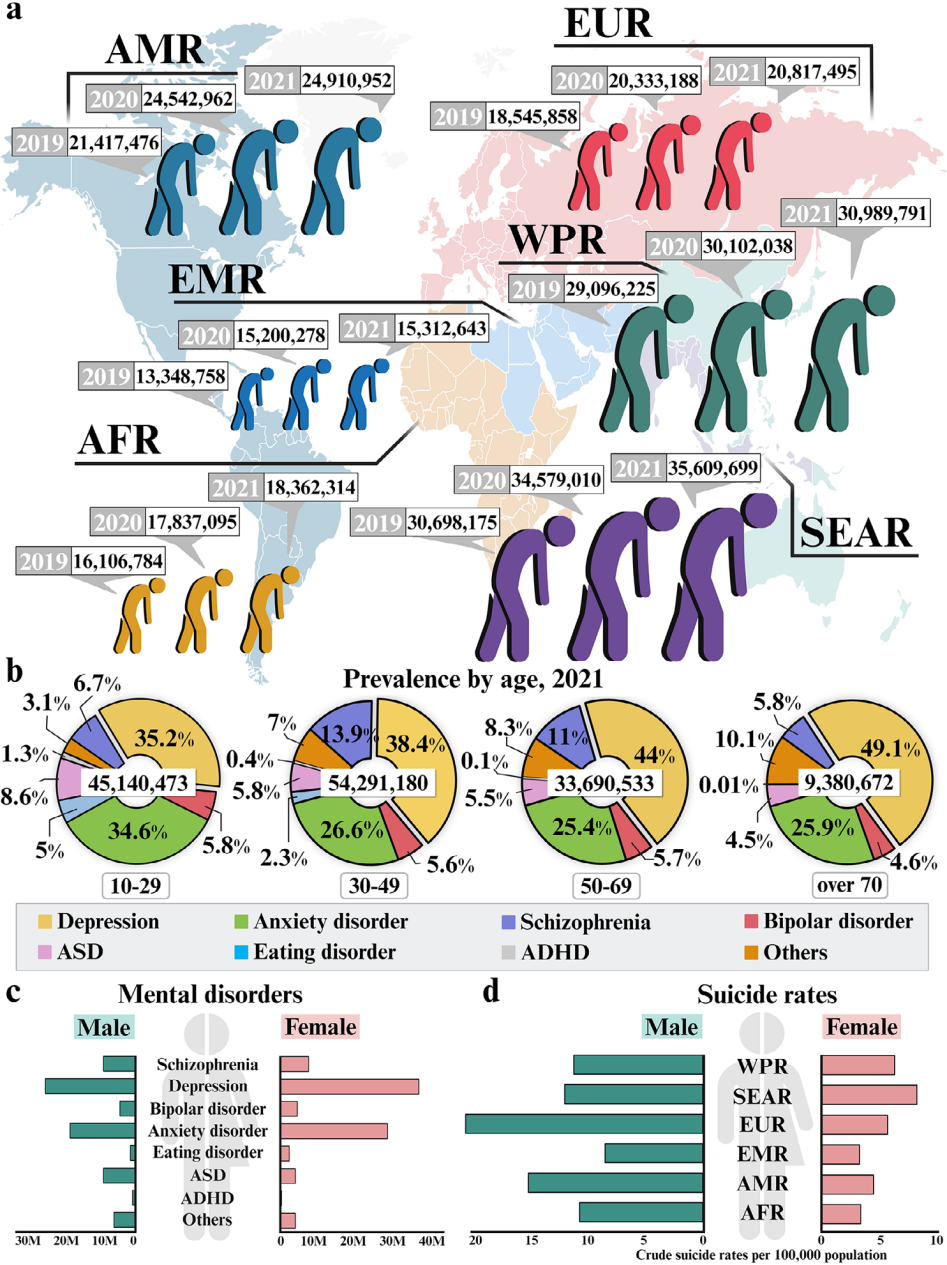
Worldwide trend of mental disorders with suicide. a) Global map showing the incidence of mental disorders across WHO regions from 2019–2021. Each color of the graph represents a different WHO region: the Americas (AMR), African region (AFR), European region (EUR), Eastern Mediterranean region (EMR), Southeast Asian region (SEAR), or Western Pacific region (WPR). b) The global prevalence of mental disorders in 2021 exhibited different rates of representative mental disorders in an age‐dependent manner. Each pie chart represents a distinct range of ages, and each part of the graph represents the type of mental disorder. c) Both sexes at all ages revealed distinct trends in the incidence of each mental disorder worldwide in 2021. The bar graphs show the different incidence rates between males and females, according to each type of mental disorder. d) The suicide rates revealed different trends depending on sex in WHO regions in 2019. The bar graphs show the distribution of suicide rates according to WHO region and sex. Global statistical information and methods for visualizing the relationships between age and sex in individuals with mental disorders are available online from the Global Health Data Exchange and the WHO. ASD: autism spectrum disorder; ADHD: attention‐deficit hyperactivity disorder. The figure was created with BioRender.com.

Mental disorders are generally defined as psychiatric dysfunctions that are widely associated with psychological, neurological, and behavioral disorders. Various types of mental disorders, such as anxiety, anorexia nervosa, ASD, MDD, posttraumatic stress disorder (PTSD), and substance use disorder (SUD), which cause distress or dysfunctions in mental functioning, including cognition, emotion regulation, and behavior with social, occupational, or other individual activities, are diagnosed.^[^ ^1^ ^]^ According to Centers for Disease Control and Prevention (CDC) data from 2024, rates of mental health treatment have continuously increased among adults aged 19.2% to 23.9% from 2019–2023. Similar trends have been shown from statistics reports to be growing constantly among all groups by age: 18.5% to 26.6% in those aged 18–44, 20.2% to 22.6% in those aged 45–64, and 19.4% to 20.3% in those aged over 65.^[^ ^2^ ^]^ In addition, the World Health Organization (WHO) depicts one out of every eight people worldwide suffering from mental illness, such as anxiety, depression, trauma, and dissociability, which increases the risk of suicide through suicidal ideation, plans, and attempts (**Figure** **1**). Since 2020, eclipsing the COVID‐19 outbreak and lockdown has increased mental susceptibility, leading to significant suicidal ideation, and has also affected the global prevalence of long‐lasting depression and anxiety.^[^ ^3^ ^]^ These findings appear to be even worse in individuals who have already suffered from mental illness, increasing their suicidal behaviors.^[^ ^4^ ^]^ Regardless of the pandemic, the number of people coping with mental problems during their lifespan is steadily increasing, reflecting socioenvironmental factors, such as multifactor stress, single‐person households, and disparities.^[^ ^5^ ^]^ Psychiatric symptoms are common and severe due to clinically significant impairments in disability. All countries face this public health challenge, which encompasses the global burden of supporting and solving it.^[^ ^6^ ^]^

Recently, interest in the medicinal applications of psychedelics to treat mental disorders has been renewed through noticeable clinical efficacy, and multiple million dollars have been funded for psychedelics.^[^ ^7^ ^]^ Psychedelic drugs are known to be serotonergic hallucinogens in psychoactive substances that can be differentiated into two types: naturally produced psilocybin, psilocin, and *N*,*N*‐dimethyltryptamine (DMT) and synthesized LSD, *N*‐methyl‐3,4‐methylenedioxyamphetamine (midomafetamine, MDMA), 2,5‐dimethoxy‐4‐iodoamphetamine (DOI), and ketamine.^[^ ^8^ ^]^ Psychedelics generally work on 5‐hydroxytryptamine receptors (5‐HTRs), which are the targets of conventional antipsychotics and provoke hallucinations and changes in mood, perception, and cognition.^[^ ^9^ ^]^ Psychedelic studies have suggested strengths of cognition, brain connectivity, subsequent neuroplasticity, and neuronal regeneration.^[^ ^10^ ^]^ A recent case report revealed that the mental sequelae of COVID‐19 symptoms were attenuated after psychedelic treatment, suggesting its effectiveness as a mental health treatment option.^[^ ^11^ ^]^ The properties of psychedelics in neuropsychopharmacology allow them to be considered contributable therapeutics for mental disorders.

Despite the sensational clinical outcomes of psychedelics, it is not yet possible to universalize them owing to a lack of safety and efficacy. Psychotomimesis and potential abuse are drawbacks of present‐day psychedelics that should be strictly regulated for nonmedical purposes. Moreover, unclear and missing mechanisms limit medical application to distinct sexes and populations, and patients may respond differently to equal psychotherapy.^[^ ^12^ ^]^ Several shortcomings must be overcome when addressing these challenges. First, the placebo limitation makes it difficult to find trustworthy controls for blind tests. Acute dissociation after effective psychedelic treatment allows participants to notice the placebo distinguishable, which is a trip‐like acute psychedelic experience of insanity, troubling vision, fear, and paranoia.^[^ ^13^ ^]^ In contrast, clinical trials adopt mild‐trip inducible placebo, which is unexpected, unpleasant, and empirical in that some patients never want to go through again.^[^ ^13^, ^14^ ^]^ Second, there is insufficient understanding of real‐world requests for increased psychedelic usage. According to the Global Psychhedelic Survey (GPS), the responses of 6379 adult consumers from 85 countries revealed significant region‐specific patterns in hallucinogen use, frequency, and motivation for its use and dosing.^[^ ^15^ ^]^ Legal restrictions hinder the evaluation of psychedelics in many countries^[^ ^16^ ^]^ and hamper posttrial care provision, which may provide insight into psychedelic therapy.^[^ ^17^ ^]^ Loosening restrictions on drug use for life improvement is now considered necessary, as is ensuring legal access with quality control.^[^ ^15^ ^]^

To overcome the current limitations in using and investigating psychedelics, researchers have attempted to visualize the brain or retrospectively study mental disorders before and after therapy. Cerebrospinal fluid (CSF) and blood analyses allow us to deduce psychophysiological conditions through noninvasive methods in a retrospective manner, including cell counts, the presence of albumin in the CSF, and altered immunoglobulin fraction levels.^[^ ^18^ ^]^ The brain is a highly complex organ that affects systemically; therefore, researchers are striving to understand the transcriptional regulation underlying mental disorders as well. In this review, we concisely underline representative psychedelics and the current viewpoints of advanced research in drug discovery.

## 2 Current Therapeutic Approaches for Psychedelic Drugs in Mental Disorders

**Figure 2:**
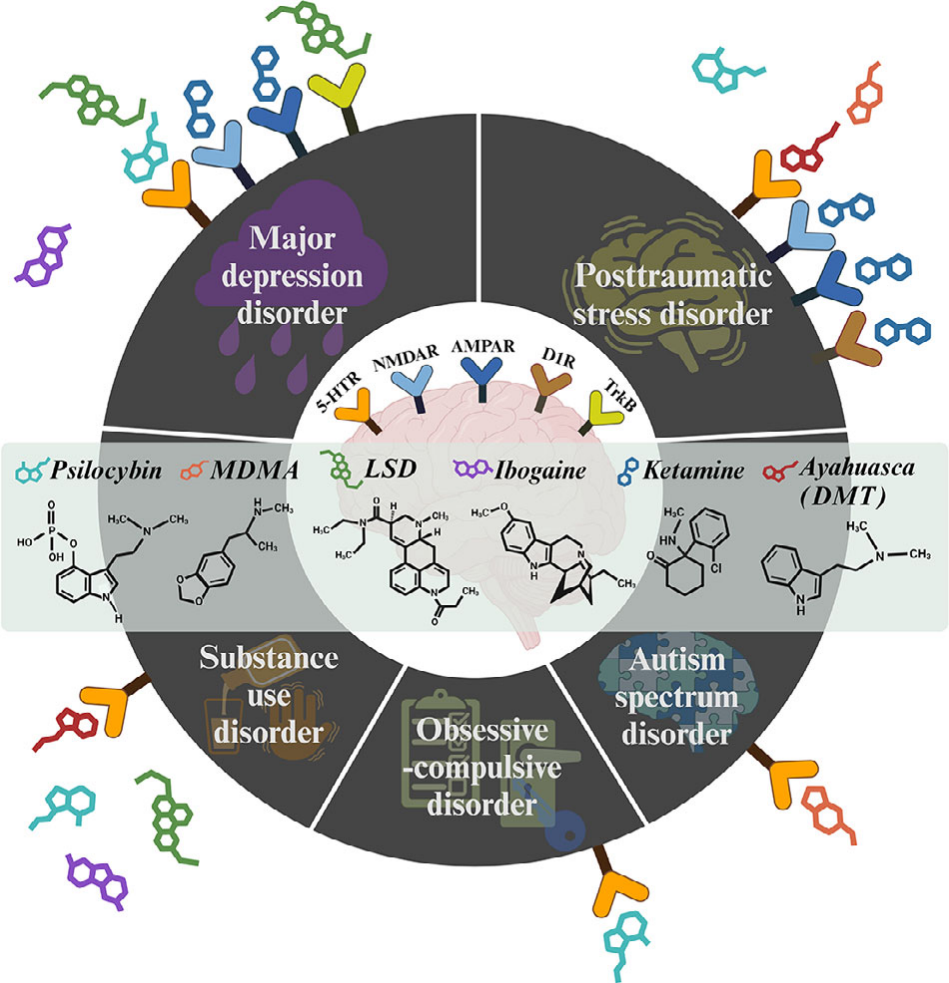
Overview of psychedelic drugs and receptors in mental disorders. Psychedelic drugs, including psilocybin, MDMA, LSD, ketamine, ibogaine, and ayahuasca (DMT as an active component in ayahuasca brew), have renewed interest in treating psychiatric disorders. Emerging psychedelics are harnessed to restore symptoms against poor response rates to traditional medications, such as major depressive disorder (MDD), posttraumatic stress disorder (PTSD), obsessive‒compulsive disorder, autism spectrum disorder (ASD), and substance use disorders (SUD) (e.g., alcohol and opioid). Some psychedelics interact collectively with diverse types or subtypes of receptors, and vice versa, owing to the structural similarities of cognate neurotransmitters. The psychedelics confer potentiation through their engaged receptors in a wide spectrum of improved outcomes. The interactive mechanism between psychedelics and receptors in response to mental disorders is still unclear and requires an explanation of which drugs are best suited to initiate a cascade. 5‐HTR: 5‐hydroxytryptamine receptor; AMPAR: α‐amino‐3‐hydroxy‐5‐methyl‐4‐isoxazolepropionic acid receptor; D1R: dopamine D1 receptor; TrkB: tropomyosin receptor kinase B; MDMA: N‐methyl‐3,4‐methylenedioxyamphetamine; LSD: lysergic acid diethylamide; NMDAR: N‐methyl‐D‐aspartate receptor. The figure was created with BioRender.com.

Psychedelic drugs have been explored to treat devastating psychiatric disorders in which profound changes in mood and alleviating outcomes occur upon psychedelic‐assisted therapy. Here, a very recent view of the respective psychedelics may provide insight for further research accompanied by experiments in rodent models (**Table** **1**) and clinical trials in patients (**Table** **2**) with mental disorders (**Figure** **2**).

**Table 1:** Psychedelic research in a rodent model.

Mental disorders	Molecular mechanism (Brain region)	Used agonist or antagonist	Dose and Route (Schedule)	Rodent model	Disease modeling	Drug treatment effect	Ref.
*Psilocybin*							
Depression	5‐HT2AR	Ketanserin	1 mg kg^−1^, IP (Single)	C57BL/6 mice	Chronic multimodal stress protocol	Hedonic responses of higher willing to choose sucrose and female urine. 5‐HT2AR activation independent.	[19]
	5‐HT2AR		0.05 mg kg^−1^, IP (1/d for 6d)	C57BL/6 mice	5‐HT2AR‐deficient	Appeared antidepressant willing to feed, swim, and choosing sucrose.	[20]
	–		1 mg kg^−1^, IP (Single)	C57BL/6 mice	Learned helplessness upon inescapable footshock Thy1‐GFP M: Tg(Thy1‐EGFP)MJrs/J	Enhanced avoidance action against footshock. Improved formation and density of dendritic spine, lasted a month.	[21]
	–		0.3 mg kg^−1^, IP (Single)	Wistar Han rats, Wistar Kyoto rats	Endogenous depressive model	Improved in novel object recognition, social interaction, and immobility. Altered neurotrophin, cognate receptor, and synaptic plasticity related gene expression level.	[22]
Anxiety	–		3 mg kg^−1^, IP (Single)	C57BL/6 mice	Chronic corticosterone exposure	Postacute subsequent elevation of glucocorticoid level in plasma.	[23]
PTSD	–		0.1–2.5 mg kg^−1^, IP (Single)	C57BL/6 mice	CS tone (80 dB, 5 kHz, 30 sec) followed by paired US footshock (0.8 mA, 2 sec)	Enhanced dendritic complexity. Fear extinction observed in lower freezing proportion.	[24]
	–		1 mg kg^−1^, IP (Single)	C57BL/6 mice	CS tone (75 dB, 4 kHz, 25 sec) followed by paired US footshock (1 mA, 2 sec)	Raised fear extinction rate after the shock omitted. Altered neural plasticity upon fear conditioning stages with calcium regulation.	[25]
Obsessive‐compulsive disorder	5‐HT2AR, 5‐HT2CR	M100907, SB242084	0.5–2 mg kg^−1^, IP (Single)	Female NMRI mice	Citalopram treatment	Attenuated obsessive symptoms observed in reduction of digging buried marbles for 30 min.	[26]
	Occupancy ratio of 5‐HT1AR	WAY100635	4.4 mg kg^−1^, IP (Single)	ICR mice	Selected mice; buried at least 15 marbles	Attenuated obsessive symptoms observed in 32.84% lowered marble‐burying behavior.	[27]
SUD	–		0.1–2 mg kg^−1^, IP (Single)	C57BL/6 mice	Two‐bottle choice drinking paradigm	Decreased dose‐dependent alcohol consumption and preference. Effect sustained for 3 days at dosage above 0.5 mg kg^−1^.	[28]
	–		1 mg kg^−1^, SC (Single)	Wistar rats	Alcohol drinking and deprivation cycle for 12 months	Reliving DMN connectivity in medial prefrontal regions led alcohol relapse intensity.	[29]
Anorexia nervosa	5‐HT2AR, 5‐HT1AR (Medial prefrontal cortex)		1.5 mg kg^−1^, IP (Single)	Sprague‒Dawley rats	Trained running wheel activity with food access restriction.	Reversed trained experience of eating, 5‐HT2A/1AR dependently. Alterations of related mRNA level in medial prefrontal cortex.	[30]
*MDMA*							
PTSD	–		7.8 mg kg^−1^, IP (single) 1 µg, BLA/IL infusion (Single)	C57BL/6 mice	CS tone (75–80 dB, 4.5 kHz, 30 sec) followed by paired US footshock (1 mA, 2 sec)	Increased locomotor activity after IP treatment. Reduced freezing ratio after BLA/IL treatments.	[31]
	5‐HTT		7.8 mg kg^−1^, IP (Single)	C57BL/6 mice	CS tone (70–85 dB, 12 kHz, 1 min) followed by paired US footshock (0.4 mA, 0.25 sec)	Acute and chronic treatment of 5‐HTT inhibitor increased freezing time. (5‐HTT inhibitor interrupted MDMA action)	[32]
	–		0.01–10 mg kg^−1^, IP (Single)	C57BL/6 mice	CS tone (75–80 dB, 4.5 kHz, 30 sec) followed by paired US footshock (1 mA, 2 sec)	10 mg kg^−1^kg^−1^ increased locomotor, stereotyped, and vertical activity of behavioral sensitization, and conditioned place preference and responding. 3 or 10 mg kg^−1^ decreased immobility time in FST. (Amnesia‐induced at high dose (> 3 mg kg^−1^))	[33]
	5‐HT1AR, 5‐HT2AR, Glucocorticoid receptor	Pindolol, ketanserin, mifepristone	5 mg kg^−1^, IP (Single)	Sprague Dawley rats, Lewis rats	Predator‐scent stress	Ameliorate stressful reaction.	[34]
Prosociality	5‐HT1BR	NAS‐181	7.5 mg kg^−1^, IP (Single)	C57BL/6 mice	Genetic manipulation *SERT, OxtR*	Prosociality increased with rewarding‐like additive liability via place preference test. Increased exploring time to center chamber of the three‐chamber test.	[35]
ASD	5‐HT1BR (NAc)		7.5 mg kg^−1^, IP (Single)	C57BL/6 mice	Genetically defected on *chr16p11.2, Arid1b*. Environmental manipulation via injection VPA sodium salt to pregnant mouse.	Elevated social interaction ratio. Increased exploring time in the center chamber of the three‐chamber and juvenile interaction tests.	[36]
	–		7.8 mg kg^−1^, IP (1/w or 1/3d)	Male Swiss Webster mice	Sensitization	Prefer to explore a novel conspecific, whereas 5‐HT2AR inhibited mice were limited.	[37]
*LSD*							
Anxiety	–		5–30 µg kg^−1^, IP (1/d for 7 d)	C57BL/6 N mice	Chronic restraint stress induced 2 hours/day for 15 days.	Preventing stress‐induced anxiety symptomatic behavior at 30 µg kg^−1^.	[38]
SUD	–		50 ug kg^−1^, IP (Single)	C57BL/6J mice	Two‐bottle choice drinking paradigm	Decrease in alcohol consumption in both water and 20% ethanol in their cage.	[39]
	–		0.1 mg kg^−1^, IP (Single)	C57BL/6JRj mice	Consume 20% ethanol for 2 hours for 4 weeks	Reduced intermittent ethanol intake.	[40]
Prosociality	5‐HT2AR, AMPAR (Medial prefrontal cortex)		30 ug kg^−1^, IP (1/d for 7 d)	C57BL/6J mice	Raptor^f/f^: Camk2α‐Cre	Enhanced social behavior through 5‐HT2AR‐ and AMPAR‐derived excitatory transmission that activate mTOR signaling.	[41]
Depression	TrkB		0.1 mg kg^−1^, IP (Single)	C57BL/6 N mice	Point mutation on the transmembrane domain of TrkB gene (Y433F±)	Increased neurotrophic signal and spinogenesis via TrkB dimerization.	[42]
	5‐HT1AR, 5‐HT2AR	Alpha‐MS, 8‐OH‐DPAT	0.13 mg kg^−1^, SC (1/d for 11 d)	Male Wistar rats	Bilateral olfactory bulbectomy	Successful escape and avoidance in lump‐jumping test.	[43]
	–		0.15 mg kg^−1^, IP (Single)	Wistar Kyoto rats	Endogenously depressive	Decreased depressive‐like behavior observed in reduced swimming time in FST.	[44]
*Ketamine*							
Sex‐dependent metabolism	–		10 mg kg^−1^, IP (Single)	CD‐1 mice	–	Male: Total plasma concentrations of ketamine and norketamine were higher, and (2R,6R;2S,6S)‐HNK were lower than female.	[45]
						Female: (2R,6R)‐HNK administration resulted in higher levels than male.	
MDD	GluN2B (Neocortex)		50 mg kg^−1^, IP (Single)	C57BL/6J mice	GluN2BΔCtx mice	Increased mobility time and open arm staying time, and decreased travel distance.	[46]
	GluN2A (Hippocampus)		10 mg kg^−1^, IP (Single)	C57BL/6J mice	Grina2a‐/‐ mice (GluN2A‐/‐mice)	Lowered mobility and decreased travel distance.	[47]
	5‐HTT		20 mg kg^−1^, IP (Single)	C57BL/6J mice	5‐HTT KO	Lowered mobility and locomotor activity.	[48]
	–		0.1 cc/10 mg, IP (Single)	129S6/SvEv mice	Contextual fear conditioning; 3‐shock at 180, 240, and 300 sec (2 sec duration each, 0.75 mA)	–	[49]
					Learned helplessness; chronic immobilization stress (70 shocks, 0.5 mA, and 15 sec interval)		
	GluN2A (Hippocampus)		30 mg kg^−1^, IP (Single)	C57BL/6J mice	CSDS induction protocol; CD‐1 breeder with aggressive behaviors attacks mice for 10 min, repeated 10 days.	Lowered escape failure.	[50]
			10 mg kg^−1^, IP (Single)			Improved memory observed from novel object recognition.	
	AMPAR	NBQX	7.5 mL kg^−1^, IP (Single)	C57BL/6J mice	–	Decreased immobility time, escape failure, and eating rejection in unfamiliar environment.	[51]
PTSD	NMDAR or AMPAR subunit (p‐GluN2B, p‐GluR1) (NAc)		15 µg µL^−1^, NAc bilateral (Single)	C57BL/6J mice	Fear conditioning; Context A, 5 presentations of two sounds (10 sec of 5 kHz clicks, or continuous 1 kHz sound with 5 ms rise and fall, 70 ± 5 dB); Context B, 5 trails each CS −/+ sounds (10 sec, average 70 sec intervals, ranging 40–100 sec)	Freezing ratio significantly decreased the in response to CS tone.	[52]
	p‐mTOR/mTOR, BDNF, and PSD‐95 (NAc)		10–100 µm, microinjection to NAc (Single)	Male Sprague Dawley rats	SPS&S induced PTSD model; immobilized for 2 hours then immediately forced swim for 20 min and 15 min break. Anesthetized until unconscious. Drug injection in 30 min followed by electric shock (4 sec, 1 mA). Processed for 14 days.	50 µm decreased anxiety‐ and depressive‐associated behaviors improving exploration, locomotor behavior and social interaction.	[53]
	ERK, D1R, and c‐fos		50 mg kg^−1^, IP (Once a day for 7 d)	Male Sprague Dawley rats	Ketamine abuse model was established administration of ketamine IP injection.	Ketamine abusing enhanced the anxious behavior and disrupted NAc^a)^	[54]
*Ibogaine*							
SUD	GDNF		10 or 30 mg kg^−1^, PO (Single)	Male Swiss mice	Operant ethanol‐paired compartment CPP	Lowered time‐gap in CPP test between two places after ethanol re‐exposure.	[55]
	–		40 mg kg^−1^, IP (Single)	Male Long Evans rats	Operant ethanol self‐administration	Reduced ethanol intake and place preference.	[56]
	–		Noribogaine: 3–300 ng/ul, 0.8 µl/injection, Intra‐VTA cannulation (Single)	Male Long Evans rats	Operant ethanol self‐administration	Lowering ethanol intake and seeking preferable place voluntarily. Both ibogaine and noribogaine upregulated GDNF in SH‐SY5Y cell line.	[57]
	–		10–100 mg kg^−1^, PO (Single)	Swiss Webster mice, Sprague Dawley rats	Addicted to morphine via dose escalation (SC injection)	Abolition against opiate dependence; reduced body and foot tremors, jumping, and diarrhea.	[58]
	–		50 mg kg^−1^, SC (Single)	Male Sprague Dawley rats	Morphine conditioning	Upregulated myelin in white matter of brain.	[59]
	–		Noribogaine: 12.5–50 mg kg^−1^, PO (Single)	Male Sprague Dawley rats	Trained nicotine self‐administration by pressing the lever.	Reduced seeking nicotine. Similar level to smoking cessation drug varenicline treatment.	[60]
Depression	–		Ibogaine, noribogaine: 20 or 40 mg kg^−1^, IP (single)	Male Wistar rats	Stress and sensitize the animals to develop immobility for 15 min.	Reduced immobilization time took 3 hours by ibogaine and 0.5 hours by noribogaine.	[61]
	–		Noribogaine: 20 or 40 mg kg^−1^, IP (single)	Sprague Dawley rats	Chronically implanted with electrodes to monitor the states of sleep and wakefulness.	Suppressed REM sleep duration rather than REM sleep initiation time. (Recorded in EMG of motor, somatosensory, and cerebellum)	[62]
*Ayahuasca*							
SUD	–		130, 1300, 2600 mg kg^−1^, IP 1300 mg kg^−1^, VO (Single)	Male Swiss mice	Ethanol induced CPP paradigm; either ayahuasca or vehicle is pretreatment, followed by ethanol treatment conditioning in 1 hour.	No influence on motor activities while inhibited the ethanol‐induced CPP. Dose at 2600 mg kg^−1^ was lethal.	[63]
	5‐HT2AR	M100907	100 mg kg^−1^, PO (Single)	Male Swiss mice	Two‐bottle choice ethanol self‐administration; acquisition, treatment, and re‐exposure to ethanol (10% v/v) for 30 days.	Ethanol self‐administration and ethanol intake behaviors are decreased which persisted during re‐exposure tests.	[64]
PTSD	–		60, 120 or 240 mg kg^−1^, PO (Single)	Wistar rats	Contextual fear conditioning; Context A, 3 electric footshocks (0.7 mA/3 sec); Context B, no reactivation experiments; Context C, single footshock (0.4 mA, 3 sec)	60 mg kg^−1^ group impaired fear memory reconsolidation by anxiolytic effect.	[65]
*DMT*							
SUD	5‐HT1AR (Striatum, Hippocampus)		1.76 mg kg^−1^, PO (Single)	Male Swiss mice	To evaluate EIBS, 2.2 g kg^−1^ ethanol or saline IP injections for odd days (Day 1–9)	Reduced locomotor activities.	[66]
PTSD	–		10 mg kg^−1^, IP (Single)	Male Sprague Dawley rats	Fear conditioning; auditory cues (80 dB white noise, 30 sec) with a foot shock (0.8 mA, 2 sec) spaced 90 sec.	Acute‐dose DMT occurred anxiogenic effects. Reduced exploratory behavior, total travel distance and the spent time in rearing or stereotypes.	[12b]
	–		1 mg kg^−1^, IP (1/3d for 2w)	Sprague Dawley rats	Fear conditioning; auditory cues (80 dB white noise, 30 sec) with a foot shock (0.8 mA, 2 sec) spaced 90 sec.	Increased total distance traveled, number and spent time of rearing, and degree of thigmotaxis. Anxiogenic effects were not observed in chronic and low doses.	[12c]
	5‐HT1AR, 5‐HT2AR (Infralimbic cortex)		0.3 mg kg^−1^, PO (Single)	Wistar rats	Contextual fear conditioning (US; 1.0 mA, 60 Hz, 3 sec, with a 30 sec intertrial period); Context A, fear extinction recall; Context B, neutral and novel context; Context C, single US; 1.0 mA, 60 Hz, 3 sec.	Contextual freezing extinction without affection recall. Anxiolytic effect was not observed.	[67]

^a)^ The effect of ketamine was added to depict the drug mechanism in detail. Sex was only denoted when references were indicated. 5‐HT1AR: 5‐HT1A receptor; 5‐HT1BR: 5‐HT1B receptor; 5‐HT1CR: 5‐HT1C receptor; 5‐HTT: 5‐HT transporter; 8‐OH‐DPAT: 8‐hydroxy‐2‐[di‐n‐propylamino] tetralin; Alpha‐MS: alpha‐methylserotonin; AMPAR: AMPA receptor; ASD: autism spectrum disorder; BDNF: brain‐derived neurotrophic factor; BLA: basolateral amygdala; CPP: conditioned place preference; CS tone: conditioned stimulus tone; CSDS: chronic social defeat stress; D1R: dopamine D1 receptor; DMN: default mode network; DMT: N,N‐dimethyltryptamine; EGFP: enhanced green fluorescent protein; EIBS: ethanol‐induced behavioral sensitization; EMG: electromyogram; EPMT: elevated plus maze test; ERK: extracellular signal‐regulated kinases; FST: forced swim test; GDNF: glial cell line‐derived neurotrophic factor; HNK: hydroxynorketamine; IL: infralimbic; IP: intraperitoneal; KO: knockout; LCA: locomotor activity; LH: learned helplessness; LSD: lysergic acid diethylamide; MDD: major VPA: valproic acid; VTA: ventral tegmental area.

**Table 2:** Psychedelic drugs in the trial.

Psychedelic	Condition	Participant group	Dose and Route	Measure		Drug effect (Allocation/Masking)	Clinical trial	Study (start‐end)	Ref.
Psilocybin	Depression	TRD patients from 10^a)^ countries (Total, n = 233)	1–25 mg, PO	MADRS	MADRS: 6.6 point ↓ (10% ↓)	25 mg group; observed relief on 3^rd^ week but did not last. (Randomized/Quadruple‐blinded)	NCT03775200^b)^	Phase 2 (2019 Mar–2021 Sep)	[68]
		TRD patients in Canada (Total, n = 30)	25 mg, PO	MADRS	MADRS: scores ↓	Immediate group showed Large Hedge's g effects 1.07 compared to waitlist, and repeated group improved greater. (Randomized/Open label)	NCT05029466	Phase 2 (2021 Nov–2023 Jul)	[69]
		TRD patients in US (Total, n = 104)	25 mg, PO	MADRS	MADRS: 12.3 points (day 43) ↓ 12 points (day 8) ↓	Reduced depressive symptoms and functional disability. (Randomized/Triple‐blinded)	NCT03866174^b)^	Phase 2 (2019 Oct–2022 Jun)	[70]
		TRD patients in US (Total, n = 225)	25 mg	MADRS, SDS	Not provided yet.	Not provided yet. (Randomized/Quadruple‐blinded)	NCT05624268^b)^	Phase 3 (2023 Jan–2025 Jul)	NA
		MDD patients in US (Total, n = 240)	5–25 mg, PO	MADRS, CGI‐S, SDS	Not provided yet.	Not provided yet. (Randomized/Triple‐blinded)	NCT06308653^b)^	Phase 3 (2024 Mar–2026 Apr)	NA
		MDD patients in US (Total, n = 57)	12 or 16 mg	MADRS	MADRS: 60% ↓ (12 mg) 75% ↓ (16 mg)	Reduced depression symptoms sustained 4‐month. (Randomized/Quadruple‐blinded)	NCT05385783^b)^	Phase 1 Phase 2 (2022 Aug–2024 Jan)	NA
	Anxiety	Patients with advanced‐stage cancer in US (Total, n = 12)	0.2 mg kg^−1^, PO	5D‐ASC, BDI, STAI, POMS	5D‐ASC: variety scores ↓ BDI: 30% ↓ STAI: 1.81 points ↓	Anxiolytic effect showed in decreasing scores in surveys and positive trends. (Randomized/Quadruple‐blinded)	NCT00302744	Phase 1 (2004 Apr–2008 Dec) Phase 2 (2022 Apr–2024 Mar)	[71]
		Patients with advanced‐stage cancer in US (Total, n = 56)	22–30 mg/70 kg, PO	GRID‐HAMD, HAM‐A	GRID‐HAMD, HAM‐A: 50% ↓	Approximately 80% of participants decreased depressed mood and anxiety. (6‐month follow‐up) (Randomized/Quadruple‐blinded)	NCT00465595	Phase 2 (2007 Apr–2016 Dec)	[72]
		Patients with psychosocial distress episode of cancer in US (Total, n = 29)	0.3 mg kg^−1^, PO	HADS, BDI, STAI	Variety items ↓	Approximately 60–80% of participants decreased in depression or anxiety. (6.5‐month follow‐up) (Randomized/Quadruple‐blinded)	NCT00957359	Early Phase 1 (2009 Feb–2018 Sep)	[73]
				DEM, HAI, DAS, DTS	Variety items ↓				
		Patients with MDD and cancer in US (Total, n = 30)	25 mg, PO	Semistructured interview	Semistructured interview: 19.1 points ↓	Reduced depression severity scores. Sustained 8 weeks without side effects. (NA/Open label)	NCT04593563	Phase 2 (2020 Sep–2023 Oct)	[74]
MDMA	PTSD	Patients with developmental trauma events, combat exposure, veteran status etc. in US, Canada and Israel (Total, n = 100)	40–80 mg, PO	CAPS‐5, SDS	CAPS‐5: 10.5 points ↑ SDS: 1.1 points ↑	Scores decreased on surveys. MDMA‐assisted therapy alleviated severe PTSD after FDA‐approved first‐line drugs sertraline and paroxetine treatment. (Randomized/Triple‐blinded)	NCT03537014	Phase 3 (2018 Nov–2020 Aug)	[75]
	Anxiety	Patients with a life‐threatening illness in US (Total, n = 18)	75–125 mg, PO	STAI	STAI: 14.7 points ↓	Reduced anxiety. MDMA was well‐tolerated in the patients. (Randomized/Quadruple‐blinded)	NCT02427568	Phase 2 (2015 May–2018 May)	[76]
		Social anxiety in autistic adult patients in US (Total, n = 12)	75–125 mg, PO	LSAS	LSAS: 1.4 scores (1 month) ↓ 1.1 scores (6 month) ↓	Scores decreased on surveys. Social anxiety symptoms demonstrated durable improvement. (Randomized/Triple‐blinded)	NCT02008396	Phase 2 (2014 Apr–2017 Apr)	[77]
LSD	Anxiety disorder	Patients in US (Total, n = 198)	25–200 µg, PO	HAM‐A	Not provided yet.	Decreased 48% clinical remission rate as anxiety symptoms. (Randomized/Quadruple‐blinded)	NCT05407064^b)^	Phase 2 (2022 Aug–2023 Nov)	NA
		Patients with severe somatic diseases in Swiss (Total, n = 46)	200 µg, PO	STAI, HAM‐D, HAM‐D‐21, BDI, SCL‐90‐R	Variety items ↓	Reduced scores of anxieties and depression. Long‐lasting reduction in anxiety and depressive symptoms over 16 weeks. (Randomized/Quadruple‐blinded)	NCT03153579	Phase 2 (2017 Jul–2021 Dec)	[78]
	PTSD	Complex PTSD patients in Swiss (Total, n = 50)	25–100 µg, PO	5D‐ASC, MEQ	Variety scores ↓	Patients with depressive symptoms decreased higher in the group setting than individual setting. Most patients answered no side effects in the surveys.	NA	(2015–2022)	[79]
	Depression	Healthy participants in US (Total, n = 80)	13 µg, PO	DEQ, ARCI, PANAS	Variety scores ↓	Depression‐related scores reduced, and minimal LSD‐induced altered brain connectivity in limbic circuits. (Randomized/Triple‐blinded)	NCT03790358	Phase 1 (2018 May–2023 Jan)	[80]
	Emotional processing	Healthy recreational psychedelic drug users in Netherland (Total, n = 24)	5–20 µg, PO	PVT, DSST, CCT, POMS, VAS, EDI, 5D‐ASC	PVT: 43% ↓ (10 µg) DSST: 45% ↓ (5 µg), 48% ↓ (10 µg) Variety items ↓	20 µg group: Positive mood, friendliness, and arousal increased. Attentional lapses decreased in surveys. (Randomized/Double‐blinded)	NTR7102	NA	[81]
	Reward process	Healthy participant (Total, n = 18)	13–26 µg, PO	RewP, LPP, FB‐P3	RewP, LPP: amplitudes ↑ (13 µg) FB‐P3: amplitudes ↑ (13 µg, 26 µg)	Enhanced reward processing by increasing the component amplitudes of three reward event‐related potentials. (Randomized/Double‐blinded)	NA	NA	[82]
Ketamine	Depression and Bipolar disorder	TRD patients aged 18–65 (Total, n = 126; MDD, n = 84; BD, n = 42)	0.5 mg kg^−1^, IV	Baseline: MADRS ≥20 or HAM‐D ≥18 Interventions: CADSS, HAM‐D, BDI	CADSS, HAM‐D: scores ↓	Characterization of the three different studies showed common result in dissociation and antidepressant response which is assessed by multiple tests. (Randomized/Quadruple‐blinded)	NCT00088699	Phase 1 Phase 2 (2004 Jul–2017 Jul)	[83]
	MDD, PTSD, ADHD	Patients from psychiatric practices located in US (Total, n = 235)	Meta‐analysis	Baseline: BDI, HAM‐A, PHQ‐9, CRS, ACE		Decreased depression‐related and anxiety‐related scores in common. (Meta‐analysis; provided chart data of KDP from KRF)	NA	NA	[84]
				Follow‐up: BDI, HAM‐A, PHQ‐9, Levine depression scale ratings					
				MEQ and EDI after KAP session					
	Depression	Treatment response patients among TRD patients (Total, n = 42; Response, n = 28)	0.5 mg kg^−1^, IV (For 40 min over 3w)	MADRS, QIDS, N‐back	Less than 50% ↓, Cognitive flexibility‐working memory and flexibility‐executive function.	Depression significantly moderated on 17^th^ week with cognition increasing. (Randomized/Single‐blinded)	NCT03027362	N/A (2017 Jan–2020 Jan)	[85]
		TRD patients (Open‐label, n = 231; Randomized double‐blind, n = 168)	R‐107 tablet; 120 mg (1/d for 8d) 30–180 mg (2/w for 9d‐12w)	MADRS CGI‐S	MADRS: −6.1 ↓	180 mg tablet group showed reducing depression gradually until 13^th^ week. (Randomized/Open label or Blinded)	ACTRN12618001042235	Phase 2 (2019 May–2021 Aug)	[86]
		TRD patients aged 18–80 in Mexico (Total, n = 41)	Esketamine 56 or 84 mg, nasal spray self‐administers (2/w for 1–4w, 1/w for 5–8w, upon clinical decision for 9w‐6 m)	EQ 5D‐5 L	Not provided yet.	6‐month follow‐up, not yet provided. (NA/Open label)	NCT04476446^b)^	Phase 3 (2020 Sep–2023 Jun)	NA
		TRD patients aged over 18 in US (Total, n = 1148)	Esketamine 56 or 84 mg, nasal spray self‐administers (For 4w)	Interview	Improvement in severity rate.	Effect of depression including emotional health, daily functioning, and social functioning were relieved. (NA/Open label)	NCT02782104^b)^	Phase 3 (2016 Jun–2022 Dec)	[87]
		TRD patients aged 18–74 in 24^c)^countries (Total, n = 676)	Esketamine 28, 56 or 84 mg, nasal spray self‐administers (For 32w)	MADRS	MADRS: scores ↓	Reduction in depressive symptoms which indicated in decreasing score from baseline at each time point. (Randomized/Open label)	NCT04338321^b)^	Phase 3 (2020 Aug–2022 Jul)	[88]
		MDD patients aged 18–65 in US (Total, n = 120, estimated)	0.5 mg kg^−1^, IV (Single)	sgACC	Not provided yet.	Not provided yet. (Randomized/Quadruple‐blinded)	NCT06213324	Phase 4 (2024 Jan–2028 Apr)	NA
		Acute suicidal depression patients aged 18–90 in US and Canada (Total, n = 1500, estimated)	0.5 mg kg^−1^, IV (For 40 min)	SSI, C‐SSRS, MADRS, WAI‐SR, BPRS, CADSS, CGI‐S, SBQ‐R, GSE‐My, PGI‐S/PGI‐I	Not provided yet.	Not provided yet. (Randomized/Open label)	NCT06034821	Phase 4 (2023 Oct–2028 Mar)	NA
		MCI‐D patients aged 50–90 in US (Total, n = 15, estimated)	0.5 mg kg^−1^, IV (Single)	MADRS, NIHTB‐CB	Not provided yet.	Not provided yet. (N/A/Open label)	NCT06069843	Phase 2 (2023 Oct–Estimated 2025 Dec)	NA
	PTSD	Patients in US (Total = 363)	0.2 mg kg^−1^ or 0.5 mg kg^−1^ or 1 mg kg^−1^, IV (Study‐dependent)	PCL‐5	PCL‐5: scores ↓	Improvement in PTSD symptoms during week 1 to 4 which showed in score reduction. (Meta‐analysis; 5 RCTs, 2 crossover trials, and 3 nonrandomized trials)	NA	(2012–2022)	[89]
		Sexual, physical and emotional abuse patients in US (Total, n = 20)	0.5 mg kg^−1^, IV (40 min infusion, TIMBER session assisted)	CAPS‐5, PCL‐5	CAPS‐5, PCL‐5: scores over 60% ↓	Persistent symptom relief showed in reduced scores of anxieties and depression. (Randomized/Double‐Blinded)	NA	NA	[90]
		Veterans aged 18–75 (Total, n = 10)	0.5 mg kg^−1^, IV (PE session assisted)	CAPS‐5, MADRS, PCL‐5, CGI‐S	Variety scores ↓	Significant score decreasing in the endpoint, showing improvement in PTSD symptoms. (NA/Open label)	NCT03960658	NA	[91]
		Veterans aged 18–75 in US (Total, n = 100, estimated)	0.5 mg kg^−1^, IV (PE session assisted)	CAPS‐5	Not provided yet.	Not provided yet. (Randomized/Quadruple‐blinded)	NCT04560660	Phase 2 (2021 Mar–Estimated 2024 Sep)	[92]
	Suicidal ideation	Patients aged 21–70 in US (Total, n = 162)	0.2 mg kg^−1^ or 0.5 mg kg^−1^ (40 min infusion, psychotherapy session assisted)	Self‐report BDI‐II GSR device.	Not provided yet.	Not provided yet. (Randomized/Quadruple‐blinded)	NCT05737693	Phase 2 (2023 Aug–Estimated 2030 Aug)	NA
		Patients aged 21–65 in US (Total, n = 30)	0.5 mg kg^−1^ (Over 40 min infusion)	HDRS‐17, SCID‐5	Not provided yet.	Not provided yet. (Randomized/Double‐Blinded)	NCT05786066	Phase 2 (2023 Apr–Estimated 2033 Mar)	NA
	Borderline personality disorder	Patients aged 21–60 with suicidal ideation in US (Total, n = 22)	0.5 mg kg^−1^, IV (40 min infusion)	MADRS, C‐SSRS, QIDS SR‐16, BDI, SDP ZAN‐BPD, SAS‐SR Short Version score, BPI, CADSS, RMET, BPRS, IAT, EEG	Variety scores ↓	Reduction of suicidal ideation severity in clinical symptoms, and improved socio‐occupational function. (Randomized/Quadruple‐blinded)	NCT03395314	Phase 2 (2018 Feb–2022 May)	[93]
	Drug‐Resistant Epilepsy	Patients age over 18 (Total, n = 8)	0.5 mg kg^−1^, IV	NDDI‐E, QOLIE‐10, GAD‐7	Not provided yet.	Not provided yet. (NA/Open label)	NCT05019885	Phase 2 (2022 Aug–2024 Dec)	NA
Ibogaine	SUD	Opioid‐dependent patients in US (Total, n = 50)	18–20 mg kg^−1^, PO	COWS, SOWS, BSCS	COWS: 4.9 scores ↓ SOWS: 10.47 scores ↓ BSCS: 4.66 scores ↓	Decreased opioid withdrawal/craving.	NA	NA	[16]
		Opioid‐ and cocaine‐dependent patients (Total, n = 191)	8–12 mg kg^−1^, PO	HCQ‐29, CCQ‐45	Variety scores ↓	Decreased opioid withdrawal/craving.	NA	NA	[94]
		Opioid‐dependent patients in New Zealand (Total, n = 14)	25–55 mg kg^−1^, PO	ASI‐Lite, BDI‐II, SOWS	Variety scores ↓	Reduced withdrawal symptoms of opioid.	NA	NA	[95]
		Opioid‐dependent patients in New Zealand and Mexico (Total, n = 44)	31.4 ± 7.6 mg kg^−1^, PO	SCQ, BDI‐II	Variety scores ↓	Patients answered a oneiric effect in survey and reduced withdrawal and craving of opioid.	NA	NA	[96]
		Opioid‐dependent patients in Netherlands (Total, n = 14)	10 mg kg^−1^, PO	SARA, DOS	Not provided yet.	Reduced withdrawal symptoms of opioid.	EudraCT no. 2014–000354‐11	(2014 Apr–2019 Oct)	NA
		Alcoholism patients (Total, n = 12)	20–400 mg, PO	Time without using alcohol, Subjective effects, Cardiovascular effects	Not provided yet.	Not provided yet. (Randomized/Quadruple‐blinded)	NCT03380728	Phase 2 (2022 Oct–2024 Dec)	NA
	PK/PD	Healthy participants in UK (Recruiting, n = 60)	20–80 mg, PO	Cmax, Tmax, AUC0‐t, AUC0‐infinity, t1/2	Not provided yet.	Not provided yet. (Randomized/Triple‐blinded)	NCT06480981	Phase 1 (2024 Jun–2024 Dec)	NA
	Trauma‐like depression	Veterans with sequelae of repeated blast exposure in US (Total, n = 30)	2–3 mg kg^−1^, PO	WHODAS, CAPS‐5, MADRS, HAM‐A	WHODAS: 30.2 ± 14.7 to 19.9 ± 16.3 or 5.1 ± 8.1 CAPS‐5: 4.8 ± 7.9 MADRS: 3.8 ± 6.0 HAM‐A: 3.9 ± 4.6	PTSD, anxiety, and depression symptoms decreased and outcomes from baseline improved in the 1‐month follow‐up cohort.	NCT04313712	Observational study (2021 Nov–2024 Feb)	[97]
Ayahuasca	SUD	Adult male with harmful alcohol consumption (Total, n = 11)	1 ml kg^−1^	SCID‐5‐CV, AUDIT, CADSS, BPRS	CADSS, BPRS: scores ↑	Reductions in alcohol use and depression at the 1‐week and 4‐week follow‐up. (NA/Single‐blinded)	Approval of local ethics committee (Resolution no. 466/12 of the national health council)	NA	[98]
	Depression	TRD patients in Brazil (Total, n = 73)	1 ml kg^−1^, PO	HAM‐D, MADRS	HAM‐D, MADRS: 50% ↓	Depression severity decreased by 50%. (Randomized/Triple‐blinded)	NCT02914769	Phase 2 (2014 Feb∼2016 Dec)	[99]
	Depression/PTSD	Healthy participants (Total, n = 12)	0.5 mL kg^−1^, 1 mL kg^−1^, 2 ml kg^−1^, PO	Safety and dose finding study of a standardized	Not provided yet.	Not provided yet. (Randomized‐blinded)	NCT05894902	Phase1 (2023 Oct∼Estimated 2024 Jun)	NA
	CMRglc	Healthy participants in Switzerland (Total, n = 12)	PO	PET	Not provided yet.	Not provided yet. (Randomized/Single‐blinded)	NCT06252506	Phase 1 (2024 Jan∼Estimated 2025 Apr)	NA
	Processing of information in the brain	Healthy participants in US (Total, n = 200)	Not provided yet.	MEG//EEG, Q‐LES‐Q, Q‐LES‐Q‐SF, SMQ, ASI, PHQ‐9, PCQ‐26	Not provided yet.	Not provided yet.	NCT06624137	(2024 Dec∼Estimated 2028 May)	NA
DMT	Emotional processing	Healthy participants from Netherlands (Total, n = 43; males = 22; females = 21)	DMT 0.6 mg kg^−1^, PO/Harmine 50 mg, PO	MET, PCT, SWLS, BFI, EQ, EDI, VAS, PEQ	Variety scores ↑	Improved cognition, empathy, satisfaction with life, personality, and psychological well‐being. (NA/Placebo‐controlled)	Ethics review committee of psychology and neuroscience and maastricht university (ERCPN‐175‐03‐2017)	2017–2019	[100]
		Healthy participants (Total, n = 30)	20 mg, IV	QIDS‐SR16, STAI‐T, NEO‐FFI, WHO‐5, MLQ, LOT‐R, GQ‐6	Variety scores ↑ (Significant improvements in scores of depressions)	Improved emotion and psychological well‐being persisted 3‐month. (NA/Single‐blinded)	Approved by the NRES Committee London‐Brent and the Health Research Authority	NA	[101]
		Healthy male participants (Total, n = 31)	MAO‐I or DMT 100 mg, intranasal	PIQ, EBI, CEQ, PEQ	Variety scores ↑	Improved psychological insights and emotional breakthroughs. (Randomized/Double‐blinded)	Approval of cantonal ethics committee of the canton of Zürich (Basec‐Nr. 2018‐01385) and Swiss federal office of public health [BAG‐Nr. (AB)‐8/5‐BetmG‐2019/008014]	NA	[102]
	PK/PD	Healthy male participant aged 20–40 in Swiss (Total, n = 10)	DMT 30–50 mg, intranasal/Harmine 250 mg, via orodispersible tablets	VAS‐scale	VAS‐scale: scores ↑	Subjective intensity, liking, and arousal increased in both forms. (Randomized/Quadruple‐blinded)	NCT04716335	Early Phase 1 (2020 Dec–2022 Jan)	[103]

^a)^ 10 countries: Canada, the Czech Republic, Denmark, Germany, Ireland, the Netherlands, Portugal, Spain, the United Kingdom, and the United States.

^b)^ FDA‐granted breakthrough therapy.

^c)^ 24 countries: Argentina, Austria, Belgium, Brazil, Bulgaria, Czechia, Denmark, Finland, Germany, Greece, Hungary, Israel, Kazakhstan, Republic of Korea, Malaysia, Netherlands, Norway, Poland, Portugal, South Africa, Sweden, Taiwan, Turkey, and the United Arab Emirates. 5D‐ASC: 5‐dimensional altered states of consciousness rating scale; ACE: adverse childhood event score; ASI: aberrant salience inventory; ARCI: addiction research center inventory; ASI‐Lite: addiction severity index lite; AUDIT: alcohol use disorders identification test; BD: bipolar disorder; BDI: Beck Depression Inventory; BFI: Big Five Inventory; BPRS: brief psychiatric rating scale; BSCS: brief substance craving scale; BSS: Beck suicide scale total score; C‐SSRS: Columbia‐suicide severity rating scale; CADSS: clinician‐administered dissociative states scale; CAPS: depression depression inventory; BFI: Big Five Inventory; CCQ: cocaine craving questionnaire; CCT: cognitive control task; CEQ: challenging experience questionnaire; CGI‐S: clinical global impression‐severity of illness scale; CGI: clinical global impressions; CMRglc: cerebral metabolic rate of glucose; COWS: clinical opioid withdrawal scale; CRS: childhood resilience scale; DAS: death anxiety scale; DEM: demoralization; DEQ: drug effects questionnaire; DOS: delirium observation scale; DSM: diagnostic and statistical manual of mental disorders;

### 2.1 Psilocybin

The natural prodrug psilocybin (4‐phosphoryloxy‐*N*,*N*‐dimethyltryptamine) is a secondary metabolite of psychedelic mushrooms that undergoes pharmacokinetic conversion via rapid dephosphorylation to bioactive psilocin.^[^ ^104^ ^]^ Both psilocybin and psilocin share structures similar to those of the neurotransmitter serotonin, which interferes with the serotonergic cascade via various 5‐HTR subtypes.^[^ ^105^ ^]^ These properties profoundly influence perception, cognitive function, and emotional regulation.^[^ ^106^ ^]^ Although the function of psilocybin is not fully understood, this psychedelic has been harnessed in psychiatry, especially for life‐threatening depressants. Psilocybin was designated a breakthrough therapy designation by the Food and Drug Administration (FDA) in 2018, 2019, and 2024 owing to its potency for monotherapy or combination treatment.^[^ ^107^ ^]^ Several types of psilocybin‐assisted therapies are currently in clinical trials.

Psilocybin was reported to alleviate depressive symptoms in a phase 2 clinical trial that included 22 sites in 10 countries with treatment‐resistant depression (TRD). Eligible participants with randomized treatment were assessed until 2021 and sponsored by the Compass Pathfinder. Changes in psilocybin endpoints were reduced according to the Montgomery–Asberg Depression Rating Scale (MADRS). All dosages demonstrated responses, remission, and sustained responses that were appraised as reduced percentages.^[^ ^68^ ^]^ Psilocybin, named COMP360 (NCT05624268), has started phase 3 clinical trials that are estimated to end in 2025. Psilocybin‐assisted therapy was further validated in NCT05029466 in a major depressive or bipolar II disorder background, in which repeated psilocybin treatment resulted in MADRS reduction.^[^ ^69b^ ^]^ In 2023, psilocybin (PSIL301) was reported to suppress depressive symptoms in a phase 2 clinical trial in a 2‐year follow‐up study of patients with MDD sponsored by the Usona Institute. Participants belonging to the United States (U.S.) at 11 sites received a 25 mg dose of psilocybin with support from therapists; MADRS scores improved with their response, remission, and sustained response.^[^ ^70^ ^]^ A psilocybin for MDD, dubbed uAspire, started a phase III clinical trial in 2024 (NCT06308653). CYB003 by Cybin Inc. developed a psilocybin‐based deuterated analog. CYB003 was started in phase 2 (NCT05385783) in 2022 to determine the tolerance of participants with MDD; nationwide phase 3 is planned to be initiated in 2024. Literature reporting CYB003 was not found; therefore, the information was obtained from Cybin. (https://cybin.com/cyb003/).

Several additional clinical trials using psilocybin to ameliorate anxiety disorders and MDD in multiple cases are ongoing. When patients cope with cancer in advanced stages and acute stress disorders concurrently, depressive symptoms are easily dominant. A phase 2 clinical trial (NCT04593563) of psilocybin in MDD reported reduced MADRS scores without significant adverse events.^[^ ^74^ ^]^ A large‐scale study (NCT00465595) reported improved mood and decreased anxiety in ≈80% of participants,^[^ ^72^ ^]^ as it was at a modest dose (NCT00957359).^[^ ^73^ ^]^ Over the past two decades, over 130 clinical trials of medication options for treating mental illness have been conducted at ClinicalTrials.gov. There has been no single drug approved by the FDA hitherto; however, three candidates are now in phase 3 trials with the aforementioned possibilities, and numerous studies are aggressively facing the challenge of overcoming lingering concerns.

### 2.2 MDMA

MDMA, alias molly or easy, is a synthesized drug that is notoriously used for recreational purposes. MDMA is an amphetamine structure‐based substituent that mechanistically participates as a monoamine‐releaser‐inducing neurotransmitter, mostly in presynaptic neurons. Serotonin, dopamine, and norepinephrine transporters account for MDMA‐preferable interactions, which lead to increased monoamine concentrations via reuptake inhibition.^[^ ^108^ ^]^ MDMA has also shown a greater binding affinity for serotonin receptors, which is especially attributed to mood changes.^[^ ^109^ ^]^

Pharmaceutical treatment with MDMA results in elevated oxytocin release, facilitating emotional processing that supports sociability.^[^ ^110^ ^]^ In 2017, the FDA granted MDMA a breakthrough therapy focusing on PTSD in Lykos Therapeutics.^[^ ^111^ ^]^ Since patients with PTSD feel numb and are less interested in others, MDMA was aimed at improving the sensation of deprivation. Patients with PTSD treated with MDMA have access to possibilities in phase 2 trials with respect to efficiency, efficacy, and safety.^[^ ^112^ ^]^ On the basis of tolerance to PTSD at the endpoint, the FDA admitted a phase III trial upon special protocol assessment. The study was designed to evaluate participants with severe and moderate PTSD via the MDMA; these participants were named MAPP1 and MAPP2, respectively. Both MAPP1 and MAPP2 yielded meaningful outcomes, with the mean severity score reduced from baseline.^[^ ^75b,c^ ^]^ In addition to PTSD tolerance, PTSD applicability has been measured in phase 2 trials. MDMA has also been used in studies on ASD to expedite its application. Two pilot studies reported that MDMA‐assisted therapy attenuated depression and anxiety in patients with ASD (NCT02008396), death, or neurological diseases with an overall low life expectancy (NCT02427568).^[^ ^76^, ^77^ ^]^

Independent FDA advisors denied approval of MDMA‐assisted therapy on June 4, 2024, which had undergone phase III trials for PTSD since 2017. The board's decision was pointed out as providing insufficient evidence to satisfy the remaining efficiency and safety concerns. As a psychostimulant cannot guarantee a blind test, an illicit MDMA history generated bias for nearly 40% of the participants.^[^ ^113^ ^]^ These allegations of the study design may lead to misinterpretation and endanger patients, outweighing its benefits. The consequent result still supports the idea that MDMA has potential; however, it is now facing many challenges.^[^ ^114^ ^]^

### 2.3 LSD

LSD is synthesized via an ergot alkaloid with an indolamine structure associated with serotonergic subreceptors. Recent LSD trials have assessed its potential for treating depression, anxiety, and alcoholism.^[^ ^115^ ^]^ LSD has shown anxiolytic effects that have reached phase 2 clinical trials, and studies have been conducted on patients with anxiety, depression, and PTSD. LSD [d‐tartrate] was designated breakthrough therapy by the FDA in 2024. Here, LSD (named MM‐120, NCT05407064) improved generalized anxiety disorder (GAD) in 200 eligible patients between 2022 and 2023 according to Mind Medicine. Anxiety symptoms were alleviated by 48%, showing robust efficacy, with only accessible information on the website of the sponsor at mindmed.co. Phase 3 is under preparation. Another clinical trial (NCT03153579) was conducted at the University Hospital in Basel until 2021 on anxiety symptoms. This study targeted patients with fatal mental illness, and LSD scores continuously decreased, indicating a reduction in anxiety and depression.^[^ ^78^ ^]^ To reduce the hallucinogenic effect and maintain therapeutic efficacy, the microdosing of LSD has been considered one of the answers. Healthy participants randomly administered low‐dose LSD (NCT03790358) were determined to have a positive correlation between amygdala‒middle frontal cortex connectivity and increases in positive mood measured by cerebral fluid.^[^ ^80^ ^]^ LSD, through dopaminergic neurons, is associated with reward processing. Healthy young adult participants who received LSD showed increased amplitudes of three compensatory events, reflecting an increase in the reward processing of low‐dose LSD.^[^ ^82^ ^]^ Similarly, LSD affects certain disorders by processing emotions, and minimized LSD changes emotions into a positive mood, friendliness, increased arousal, and decreased attentional lapses.^[^ ^81^ ^]^

### 2.4 Ketamine

Ketamine has been approved as an anesthetic agent, and its expanded usage in psychiatric symptoms covers interventions for TRD as well as the approval of intranasal ketamine to treat resistant unipolar depression and suicidal ideation.^[^ ^116^ ^]^ Ketamine undergoes extensive metabolic pathways via the enzymatic transformation of CYPs, which are predominantly demethylated to norketamine and then hydroxylated to hydroxynorketamines (HNKs) as a consequence. (R,S)‐Ketamine is a racemic mixture of R and S isomers, each of which has been shown to contribute differently to psychological symptoms.^[^ ^117^ ^]^ Ketamine is similar to classical psychedelics, although it predominantly acts on glutamatergic receptors rather than serotonergic receptors. Ketamine has been harnessed in more than 1500 studies at clinicaltrials.gov to treat depression.

Phase 1 and 2 trials have demonstrated the acute antidepressant effects of ketamine, as have large‐scale phase 3 trials.^[^ ^118^ ^]^ Continuous research has demonstrated the efficacy and safety of ketamine in treating depression.^[^ ^119^ ^]^ TRD patients who were administered ketamine experienced an improvement in depression severity of over 50%, with significant sustainability in learning and memory (NCT03027362).^[^ ^85^ ^]^ The effect of ketamine treatment on cognitive outcomes has been confirmed in patients with depression (ACTRN12616001096448),^[^ ^120^ ^]^ and ketamine dosing for mild cognitive impairment and depression is currently ongoing (NCT06069843). In a study conducted to verify the practical potential of oral ketamine administration in patients, R‐107 tablets were evaluated in a phase 2a trial (ACTRN12618001042235). The lowest relapse and highest remission rates were observed in follow‐up studies, with minimal dissociation and sedation effects.^[^ ^86^ ^]^ Esketamine (S)‐ketamine) through nasal spray, an emerging method for treating depressive disorders, was studied long‐term to assess its safety twice per week in a phase III trial (NCT02782104). Monitoring throughout the study reported tolerability with early mental health satisfaction, but adverse events led to the discontinuation of esketamine therapy in several participants.^[^ ^87^ ^]^ Esketamine nasal spray combined with selective serotonin reuptake inhibitor (SSRI) treatment has been suggested strategically for rapid and long‐lasting effects on depressive episodes in phase 3b (NCT04338321). Cotreatment with self‐administered nasal spray and combination treatment has been shown to result in enhanced remission of treatment‐resistant depression.^[^ ^88^ ^]^ Combinatorial therapeutics that use prolonged exposure (PE) with ketamine have been demonstrated to decrease the severity of chronic PTSD symptoms.^[^ ^91^ ^]^

Advanced investigations to access comprehensive information on ketamine are in phase 4 clinical trials to compare it with other drugs or reveal additional roles. Neural circuit mechanisms in the subgenual anterior cingulate cortex (sgACC), which regulate emotions, are currently being studied in eligible patients with MDD (NCT06213324). Compared with electroconvulsive therapy (ECT), ketamine is the most effective strategy for treating treatment‐resistant depression. As ketamine was noninferior to ECT in phases 2/3 (NCT03113968),^[^ ^121^ ^]^ a further comparative study (NCT06034821) was conducted for acute suicidal depression treatment across the lifespan.

### 2.5 Ibogaine

Ibogaine, which contains abundant alkaloids found in West Africa, is extracted from the roots of the shrublet *Tabernanthe iboga*.^[^ ^122^ ^]^ Ibogaine and its metabolite, noribogaine, transduce signals through multiple receptors, including opioids, neurotrophic factors, and dopaminergic and serotonergic receptors.^[^ ^123^ ^]^ Abolishing hallucinogenic effects and toxicity have led to the synthesis of ibogaine analogs,^[^ ^124^ ^]^ especially with respect to the addictive consumption of substrates and depressive symptoms.^[^ ^125^ ^]^ Although considerable toxicity has been reported with ibogaine, research has shown promise in opioid use disorder treatment, as measured in multiple observational studies and questionnaires. Opioid use disorder detoxification was carried out via an ibogaine treatment protocol, which involved close monitoring of withdrawal symptoms and substance craving.^[^ ^16^ ^]^ Patients seeking opioids or cocaine ameliorated their negative emotions, drug desire, compulsivity, and expectancy upon ibogaine administration,^[^ ^94^ ^]^ and a 12‐month follow‐up study reported withdrawal effects.^[^ ^95^ ^]^ Anti‐addictive drugs for alcoholism are also undergoing clinical trials (NCT03380728). However, the safety evaluation (EudraCT no. 2014–000354‐11) of ibogaine still reported prolonged QTc and bradycardia.^[^ ^126^ ^]^ Therefore, a study (NCT04313712) to treat traumatic brain injury in 30 male special operations veterans used ibogaine and magnesium together to alleviate cardiac abnormalities. Cotreatment with ibogaine and magnesium significantly reduced PTSD, anxiety, and depression symptoms, and the outcomes from baseline to the 1‐month follow‐up investigation revealed significantly decreased scores and continuous improvement.

Psychiatric disability is a conspicuous burden of social deprivation that has led to lingering mental exhaustion, both within individuals and communities, specifically after the extreme tiredness of COVID‐19. Changing the treatment paradigm beyond the current medication is now focused on classic psychedelics. Almost three decades ago, these psychedelics were halted, but numerous studies worldwide are now suggesting promising outcomes with powerful possibilities along with assisted therapy. Comparative subjective experiences seem to vary so‐called “afterglow” and are measured via a psychometric evaluation of altered consciousness, suggesting questions from a multidimensional view before taking psychedelics to real medication.^[^ ^127^ ^]^ Exploded publications, as well as many clinical trials at clinicaltrials.gov for each drug, were retrieved from PubMed.^[^ ^128^ ^]^ Mental health disorders are receiving considerable attention owing to the widespread mental instability, insecurity, and affliction that may occur in one quarter of the population. Further research on precise psychedelic applications in psychedelic‐assisted mental health is needed.

### 2.6 Ayahuasca

Ayahuasca is a decoction known to contain psychoactive chemicals extracted from *Psychotria viridis* and *Banisteriopsis caapi* from the Amazonian plant tisane.^[^ ^129^ ^]^ As hallucinogenic tryptamine, DMT is a key component of the ayahuasca beverage, which intensively alters neurological conditions such as the interpretation of sensory information.^[^ ^130^ ^]^ Ayahuasca is a Schedule I substance that is permitted only for research; however, its clinical use beyond the hallucinogenic properties of DMT has increased interest in addressing depression. Studies have shown that depression affects behaviors related to mood, anxiety, cognitive function, and sociability in rodents, which attenuate depressive symptoms as a consequence.^[^ ^12c^ ^]^ Ayahuasca or DMT alone has been administered to rodent models to assess their therapeutic and functional involvement in psychiatric symptoms, which allows us to understand their beneficial effects on depression, anxiety, substance dependency, and PTSD.^[^ ^12^, ^63^, ^64^, ^65^, ^66^, ^67^ ^]^

Patients with psychiatric symptoms of depression were evaluated with neuropsychological tests after ayahuasca administration (NCT02914769), which resulted in a rapid antidepressant effect,^[^ ^131^ ^]^ and it further attenuated suicidality.^[^ ^132^ ^]^ The formulation of ayahuasca as an analog (SM‐001) was assessed via biomarkers in the blood of healthy participants (NCT05894902). The systemic metabolism of ayahuasca after administration is also considered an aspect that has yet to be studied. The cerebral metabolic rate (CMRglc) of DMT with harmine, which is known as another component of ayahuasca, has been investigated through positron emission tomography (PET) imaging in healthy participants (NCT06252506), which is estimated to be completed in 2025 Apr. Serotonergic psychedelics are also known to positively affect well‐being. Ayahuasca and DMT were administered to the healthy or neurologically disordered groups, which were then followed up for short or longer terms. Electroencephalography (EEG) or magnetoencephalography (MEG) is used to evaluate whether drugs alter how information in the brain is processed when playing online computer games. This observational study started in 2024 Dec and is estimated to be completed in May 2025 (NCT06624137).

## 3 Multifaceted Perspectives of Drug Discovery in Psychedelics

**Figure 3:**
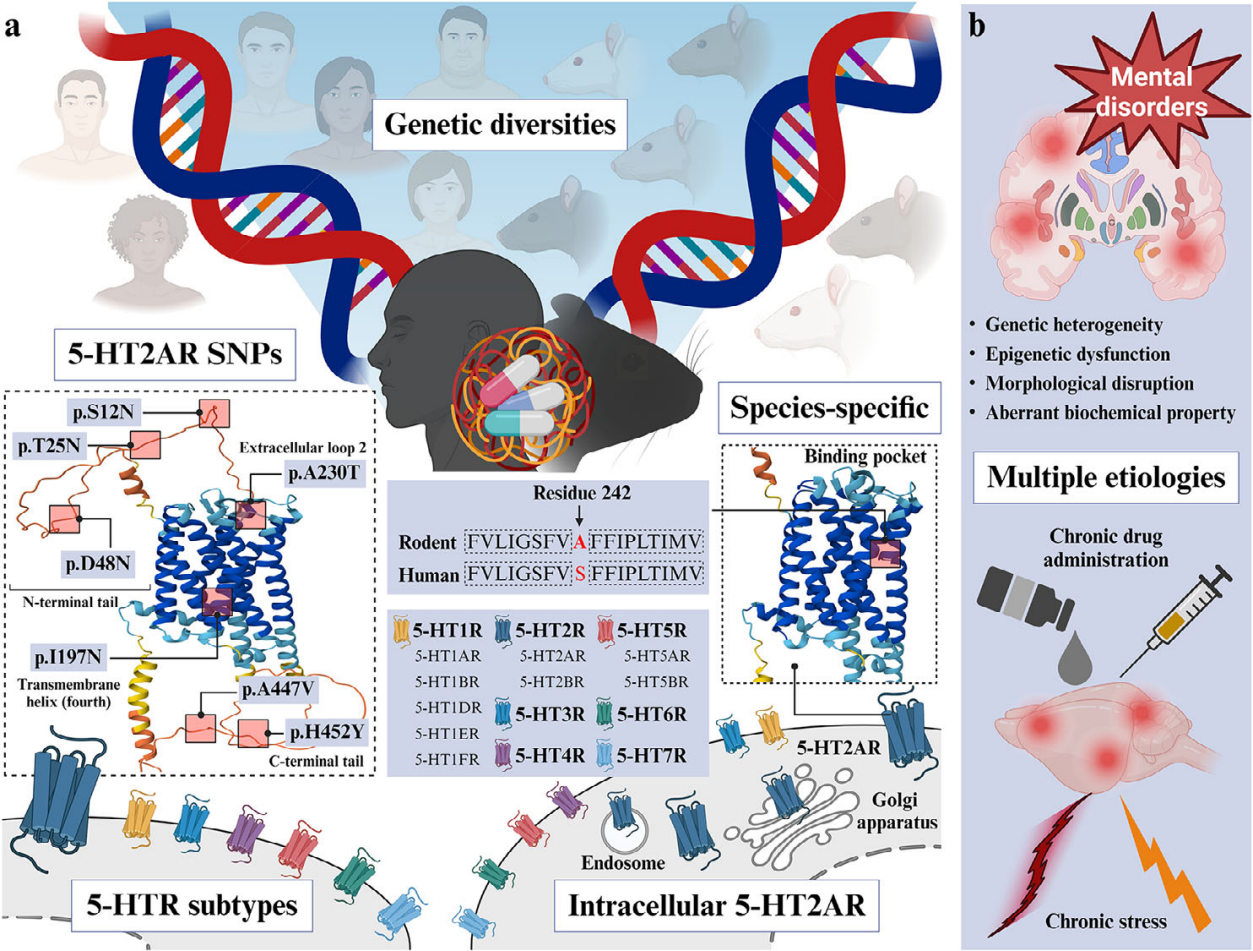
Limitations of psychedelic drugs from unintegrated pharmacological landscapes. Multifaceted strategies for evaluating the effects of psychedelic drugs on mental disorders have resulted in positive prospects for therapeutic applications. However, several challenges limit translational studies interpreting physiological outcomes after psychedelic drug treatments. a) Effects of equal psychedelic drugs largely differ in terms of genetic diversity within species, subspecies, races, and sexes. The 5‐hydroxytryptamine 2A receptor (5‐HT2AR) has a species‐specific residue 242 between human (serine) and mouse (alanine) residues in the binding pocket, as well as seven variations (p.S12N, p.T25N, p.D48N, p.I197N, p.A230T, p.A447N, p.H452Y) in human 5‐HT2AR. These changes may modify the pharmacological interactions between receptors and psychedelic drugs. Nonconserved structures may confer distinct pharmacological action across 5‐HT isoform‐dependently, where psychedelics interact. b) Rodents employed to assess the effectiveness of psychedelic drugs behaviorally and biochemically need the prerequisite induction of psychiatric symptoms in multiple ways. The relevance of behavior to human mental disorders varies with the innate properties of rodents. The diverse etiologies of mental disorders can affect the efficiency of individual differences, and mimicking the psychiatric symptoms of humans is often obstructed by orthologous differences. The figure was created with BioRender.com.

As candidate therapeutic drugs ameliorate various psychiatric symptoms according to multiple lines of evidence; however, most of them are effect‐focused to be official drugs for mental disorders, so multifaceted aspects that have been revealed in the sequential results of recent studies need to be considered.^[^ ^133^ ^]^ Toward comprehensive drug discovery, the field requires interpretation of the results from the limited‐scale participants from the specific factors comprising race and sex, genetic diversity, disorder‐stage, experience of the psychedelics, and experimental design–dose and administration route (**Figure** **3**). Therefore, to determine whether psychedelic drugs are eligible for treatment, further strategic studies are needed to establish psychopharmacological applications.

### 3.1 Structural Aspects

#### 3.1.1 Intracellular 5‐HT2AR

5‐HT2AR activation has been suggested as an important pathway in the pharmacological roles of psychedelic drugs, such as psilocybin, LSD, and DMT. However, the neurobiological mechanisms by which 5‐HTR ligands promote functional neuroplasticity are still unclear.^[^ ^134^ ^]^ In 2023, several studies reported that cytosolic 5‐HT2AR and psychedelic drug interactions increase neuroplasticity. This intracellular 5‐HT2AR has been identified in several organelles, especially those localizing to the endosome and Golgi apparatus.^[^ ^135^ ^]^ Although serotonin is known to activate 5‐HT2AR as a balanced agonist, serotonin does not induce neuronal plasticity, unlike psychedelic drugs in cortical neuron cultures.^[^ ^136^ ^]^ This is caused by the difference in membrane permeability between serotonin and psychedelic drugs, in which serotonin has an unmethylated amino group, whereas psilocin and DMT have a methylated structure. The N‐methylation structure of the psychedelics reduces the polarity and can increase the lipophilicity and membrane permeability. Psilocin, DMT, and ketamine at a dose of 1 µm enhanced neuronal growth and dendritic spine density under both the absence and presence of electroporation experimental conditions in rat embryonic cortical neurons. In contrast, membrane‐impermeable constructs of psilocybin, serotonin, and *N*,*N*,*N*‐trimethyltryptamine (TMT) allowed only neuronal growth under electroporation. Neuroplasticity in response to psilocin and DMT was suppressed by ketanserin, a membrane‐permeable 5‐HT2AR agonist, whereas methylated ketanserin only inhibited neuroplasticity via electroporation. Additionally, 10 µm serotonin treatment promoted spinogenesis and dendritogenesis, which was demonstrated via the use of the serotonin transporter (SERT) inhibitor citalopram to determine the serotonin‐induced effect on SERT‐positive neurons, whereas DMT treatment did not mediate these effects. Ketanserin abolished the neuronal growth induced by both DMT and serotonin in vitro. Compared with control mice, mice expressing CaMKII‐SERT showed an antidepressant‐like effect through treatment with para‐chloroamphetamine as a selective serotonin‐releasing agent and an increased head‐twitch response (HTR), suggesting that intracellular 5‐HT2AR activation is associated with psychedelic drugs.^[^ ^135^, ^137^ ^]^

#### 3.1.2 Pharmacological Effects with Receptors

Several lines of evidence highlight the potential participation of other critical receptors for therapeutic and/or hallucinogenic effects in psychedelics.^[^ ^138^ ^]^ For example, chronic multimodal stress paradigm (CMMS)‐induced depression mouse models were treated with a single dose of psilocybin (1 mg kg^−1^), which rapidly improved excitatory synapse and spine formation through 5‐HT2AR activation. Ketanserin pretreatment did not block psilocybin‐induced neurostructural plasticity but did reduce HTR hallucinogenic behavior.^[^ ^19^, ^21^ ^]^ Ketanserin is used to demonstrate the role of 5‐HT2AR activity in the dissociation kinetics of drugs. However, it only blocks several parts of 5‐HT2AR in rodents,^[^ ^139^ ^]^ and the ability of unblocked 5‐HT2AR to cause positive effects, including structural synaptic rewiring, could be extrapolated.^[^ ^19^, ^21^ ^]^ Additionally, several previous studies have suggested nonselective interactions between 5‐HTRs and psychedelic drugs and the possibility of binding affinity between psilocybin and other 5‐HTRs.^[^ ^26^, ^27^, ^140^ ^]^ 5‐HT1AR has been reported to interact with psilocin and DMT, but several studies have shown the opposite effect as a decrease in neuronal excitability.^[^ ^141^ ^]^ 5‐HT1BR has been reported to have beneficial effects on the behavioral and antidepressant‐associated synaptic actions of SSRIs through the activation of 5‐HT1BR, suggesting its potential beneficial effects through the interaction of psychedelic drugs.^[^ ^124^, ^142^ ^]^ In addition, other psychedelic drugs, such as LSD and DMT, have also been reported to have activity with diverse 5‐HTRs; LSD activated 11 of the 14 5‐HTRs in humans, including 5‐HT1A, 5‐HT1B, 5‐HT1D, 5‐HT1E, 5‐HT1F, 5‐HT2A, 5‐HT2B, 5‐HT2C, 5‐HT4, 5‐HT5, and 5‐HT6, as well as dopamine receptors (D1, D2, D3, and D4), adrenergic receptors (α1 and α2) and trace amine‐associated receptors (TAAR).^[^ ^134^, ^140^ ^]^ In 2024, 5‐HT2AR^−/−^ chronic despair model (CDM) mice did not exhibit HTR in response to a single dose of psilocybin, DOI, or lisuride (1 mg kg^−1^). 5‐HT2AR^−/−^ CDM mice were impeded from the previous antidepression‐like effects induced by DOI‐ and lisuride‐mediated 5‐HT2AR activation. In contrast, psilocybin continually acted as an antidepressant in 5‐HT2AR^−/−^ CDM mice, which explains the independence of psilocybin against 5‐HT2AR. Pretreatment with WAY‐100635 (5‐HT1AR antagonist) at 0.5 mg kg^−1^ did not suppress depressive symptoms, suggesting that 5‐HT1AR activation occurred in an activation‐independent manner. In addition, the antagonists of D1 and D2 dopamine receptors SCH23390 (0.03 mg kg^−1^) and eticlopride (0.03 mg kg^−1^) did not abolish these effects. Thus, D1 and D2 receptors are not the main pathways involved in the antidepressant‐like effect of psilocybin.^[^ ^20^ ^]^ Moreover, psilocin and LSD exhibit strong binding affinities with TrkB, which is known to cognate neurotrophin‐mediated neuronal features, suggesting that psilocin and LSD may be involved in psychedelic pharmacology.^[^ ^42^ ^]^ Current studies have debated the correlation between the use of serotonergic psychedelic drugs and their hallucinogenic effects, which may not confer therapeutic effects.^[^ ^143^ ^]^ The hallucinogenic effects remain unclear as to whether they are essential for therapeutic outcomes or unwanted side effects; thus, the identification of pivotal receptors for psychedelic drugs that function therapeutically is needed.^[^ ^144^ ^]^ To demonstrate these complicated relationships with the effects of psychedelic drugs and their unique receptors, many studies have attempted to understand the major psychopharmacological pathways involved.^[^ ^134^, ^136^, ^145^ ^]^

#### 3.1.3 Specific Residues

Changes in unique nucleotides or residues are closely related to the 5‐HT2AR structure. Residue 242 of 5‐HT2AR conforms to the binding pocket for psychedelics, where humans and pigs encode serine compared with rodents, which are commonly identified as alanines in equal positions. A mutagenesis study reported that the serine‐to‐alanine mutation at residue 242 in humans critically promotes the LSD‐driven dissociation rate without any change in 5‐HT2AR binding affinity. At least seven single‐nucleotide polymorphisms (SNPs) in human 5‐HT2AR were identified: p.S12N, p.T25N, and p.D48N with an N‐terminal tail; p.I197N with a fourth transmembrane helix; p.A230T with an extracellular loop 2 (ECL2); and p.A447V and p.H452Y with a C‐terminal tail. 5‐HT2AR‐carrying SNPs are associated with the agonistic efficacy and potency of psychedelic drugs, such as psilocin, LSD, DOI, 5‐methoxy‐N,N‐dimethyltryptamine (5‐MeO‐DMT), mescaline, and meta‐chlorophenylpiperazine.^[^ ^134^, ^146^ ^]^ Genetic polymorphisms can be identified throughout global populations, which can lead to different pharmacological effects of psychedelic drugs in individuals or populations.^[^ ^146^ ^]^

#### 3.1.4 Psychedelic Drug Analogs

The synthesis of nonhallucinogenic drug analogs on the basis of structure has been attempted to design new psychoactive substances (NPSs), similar to psychedelic drugs, that maintain therapeutic effects but exclude the deleterious side effects of hallucinations and toxicity through the binding affinity of 5‐HT2AR.^[^ ^136^, ^147^ ^]^ AAZ‐A154,^[^ ^148^ ^]^ IHCH‐7079, IHCH‐7086,^[^ ^149^ ^]^ 2‐Br‐LSD,^[^ ^150^ ^]^ ibogainalog (IBG), and tebernanthalog (TBG)^[^ ^124^ ^]^ are representative synthesized NPSs that alleviate behavioral symptoms and enhance neuroplasticity via 5‐HT2AR in individuals with mental disorders, especially depression. In 2024, TBG and IBG were reported to suppress several nicotinic acetylcholine receptors (nAchRs), γ‐aminobutyric acid type A receptors (GABAARs), and N‐type voltage‐gated calcium channels (Ca_v_2.2) in both humans and rats, suggesting that they are involved in other mental disorders, such as anxiety, depression, and addiction, as well; likewise, the contribution of iboga congeners as antineuropathic drugs is expected.^[^ ^151^ ^]^

### 3.2 Microbiota–Gut–Brain (MGB) and Metabolism Aspects

Recent studies have demonstrated the bidirectional correlations between the MGB axis and mental disorders, including depression, bipolar disorders, anxiety, schizophrenia, and stress‐related disorders.^[^ ^152^ ^]^ Serotonin is synthesized mainly in the gut and is released through enterochromaffin cells in the intestinal luminal epithelium, which causes mechanical or chemical stimulation, especially through interactions between the host microbiota and metabolome.^[^ ^153^ ^]^ Indeed, psychedelic drugs affect the distributions or activities of the gut microbiota; ketamine (2.5 mg kg^−1^) for 7 days increased *Lactobacillus*, *Turicibacter*, and *Sarcina*, whereas *Mucispirillum* and *Ruminococcus* decreased in male rats,^[^ ^154^ ^]^ and antimicrobial activity was suggested to be associated with the antidepressant effect via *Borrelia burgdorferi*, *E. coli*, *E. faecalis*, *P. aeruginosa*, *S. aureus*, *S. pyogenes*, *S. epidermidis*, *Stachybotrys chartarum*, and *Staphylococcus epidermidis*.^[^ ^155^ ^]^ MDMA altered the composition of *Proteus mirabilis* to be abundant, resulting in hyperthermia in rats,^[^ ^156^ ^]^ and psilocybin affected the constitution of the gut microbiota through *Verrucomicrobia* and *Actinobacteria* to be enriched and *Proteobacteria* to be depleted.^[^ ^157^ ^]^ Psilocybin, a tryptamine indole‐based monoamine alkaloid, is metabolized to psilocin as a biologically active structure, where alkaline phosphates dephosphorylate psilocybin in the intestine or acidic conditions in the stomach. Psilocin can cross the blood–brain barrier (BBB). In addition, psilocin undergoes additional metabolic processes to inactivate psilocin O‐glucuronide via glucuronidation by uridine diphosphate (UDP) glucuronosyltransferases (UGT)1A10 and UGT1A9 in the small intestine,^[^ ^158^ ^]^ and glucuronidation might affect the processing of interactions with the gut microbiota.^[^ ^159^ ^]^ The metabolite psilocin‐O‐glucuronide is removed from the urine of approximately 80% of humans, whereas only 1.5% of psilocin is removed.^[^ ^160^ ^]^ The inactivation of psilocin from 4‐hydroxyindole‐3‐acetaldehyde (4‐HIA) to 4‐hydroxyindole‐3‐acetic acid (4‐HIAA) and 4‐hydroxytryptophol (4‐HTP) occurs through demethylation and oxidative deamination by monoamine oxidase (MAO) and aldehyde dehydrogenase, and these final metabolites do not interact with 5‐HTRs.^[^ ^158^, ^161^ ^]^ Additionally, psilocin has a half‐life of up to 4 hours and is enriched in the plasma approximately 20–30 min after ingestion, which implies that the time‐dependent phenotypes rely on the intrinsic physiologies of overall health conditions, age, and sex.^[^ ^162^ ^]^ The release of fatty acid amides, such as N‐oleoylethanolamide, which can activate the cannabinoid 1 receptor and transient receptor potential vanilloid 1 in the dorsal root ganglia, is reported to occur in the brain, stomach, intestines, and liver, especially the gut microbiota with Coprococcus and Eubacterium; thus, these amides reduce MAO expression and degrade several neurotransmitters.^[^ ^163^ ^]^ DMT is the representative hallucinogen of ayahuasca and has been reported to be a potent treatment for depression, PTSD,^[^ ^101^, ^164^ ^]^ harmful use of alcohol,^[^ ^63^, ^98^ ^]^ and suicidality.^[^ ^132^ ^]^ DMT has also been reported to be composed of two metabolites in the inactivated form: indole 3‐acetic acid (IAA) and DMT N‐oxide (DMT‐NO). IAA is the most common metabolite that is metabolized primarily by MAO, and DMT NO is the second most common metabolite that is independent of MAO. Ayahuasca has β‐carboline and harmal alkaloids that suppress MAO, thereby delaying drug metabolism compared with DMT alone.^[^ ^165^ ^]^ This evidence indicates that pharmacokinetic approaches combining MGB and metabolism may contribute to the pharmacological effects of psychedelic drugs in terms of intensity and duration.^[^ ^166^ ^]^ The MGB and metabolism of psychedelics may reveal important roles in interindividual variability that need further study.^[^ ^167^ ^]^

### 3.3 Transcriptomic Aspects

In 2024, the mental health associated with suicide attempts was analyzed via polygenic risk scores (PRSs) across 334706 UK Biobank participants. The number of both behavior‐related and physiological phenotypes related to genetic predisposition was greater than 200.^[^ ^168^ ^]^ Moreover, single‐cell‐based transcriptomics has allowed subtle differences in a cell‐specific manner to assess brain complexity in detail. Continuous efforts have focused on understanding spatial information genetically,^[^ ^169^ ^]^ which is expected to decipher the mechanisms of mental disorders. Moreover, several recent studies have determined the pharmacological effects of psychedelic drugs that may interact with not only behavioral changes and enhanced brain connectivity but also gene expression.^[^ ^9^, ^170^ ^]^

#### 3.3.1 Single‐Cell Transcriptome

To determine the neurophysiological impact of psychedelic drugs on several brain areas, the function of each cell type is also focused on their correlation in response to psychedelics.^[^ ^9^, ^138^, ^171^ ^]^ Psychedelic drugs directly interact and activate excitatory neurons with various receptors that induce unique and regional responses, with transcription in multiple cell types involving a subpopulation of inhibitory parvalbumin and somatostatin GABAergic interneurons, as well as nonneuronal cells and astrocytes.^[^ ^141^, ^172^ ^]^ In 2024, the orbitofrontal cortex (OFC) collected from psilocybin (1 mg kg^−1^) intraperitoneally injected mice was processed through single‐nucleus RNA sequencing (snRNA‐seq), which distinguished the 20 clusters of cells into glutamatergic, GABAergic, and other neuronal or nonneuronal cells. Compared with parvalbumin and somatostatin GABAergic interneurons, layer 5 pyramidal neurons among the clusters presented the most altered gene expression related to synaptic transmission and cell‒cell interactions. In particular, layer 5 pyramidal neurons exhibit a decrease in excitatory transmission with the excitatory postsynaptic current (EPSC) and a reduction in the expression of the *Gria1* gene encoding the ionotropic glutamate α‐amino‐3‐hydroxy‐5‐methyl‐4‐isoxazolepropionic acid (AMPA) receptor, which is associated with excitatory synaptic transmission in the central nervous system (CNS). In contrast, somatostatin‐expressing neurons were not altered, and parvalbumin‐expressing neurons also did not change excitatory transmission, but the amplitude of spontaneous inhibitory postsynaptic currents (sIPSCs) was increased. This evidence revealed that psilocybin leads to a reduction in neuronal activity and synaptic transmission in layer 5 pyramidal neurons of the OFC, which are important output neurons in the cortex. Indeed, *htr2a* knockdown in the excitatory neurons of the OFC in chronic stress‐induced mice suppressed the psilocybin‐mediated antidepressant effect, and the deletion of *htr2a* in parvalbumin‐positive neurons only fragmentarily reduced this effect.^[^ ^173^ ^]^ This approach suggests the potential of single‐nucleus and single‐cell RNA transcriptomics for comprehensive understanding by profiling individual transcriptomic cells from unique brain regions, which can provide insight into the complicated pharmacological psychedelic mechanisms associated with cell type‐specific treatment responses in the neural network of the brain.^[^ ^174^ ^]^

#### 3.3.2 Spatial Transcriptome

Several brain regions have been shown to contain *5‐HT2AR* mRNA and protein, not only in the neocortex but also in the claustrum, amygdala, nucleus accumbens, thalamus, striatum, and mammillary nucleus, as well as differently activated brain regions across connectivity.^[^ ^9^, ^175^ ^]^ Current studies have focused on the pharmacological interactions between specific receptors and particular brain regions after the administration of psychedelic drugs. Regional primary neuron culture and diverse receptor inhibition through ketanserin, NAS‐181, M100907, SB242084, and WAY100635 have revealed interactions between psychedelic drugs and several receptors in a mouse model of mental disorders (Table 1). Multimodal neuroimaging, functional magnetic response imaging (fMRI) with magnetoencephalography (MEG) and electroencephalography (EEG) have been used to identify differentially altered activity, and positron emission tomography (PET) reveals the occupancy of receptors via selective receptor inverse agonists after treatment with psychedelic drugs.^[^ ^176^ ^]^ According to the current evidence, the claustrum is elongated into a subcortical nucleus with a thin ribbon shape and is located in the lateral putamen, which has a high proportion of 5‐HT2AR and contributes to bidirectional glutamatergic interactions with the whole cerebral cortex. This region commonly participates in the binding of sensory functions, modulation of cognitive functions, and conscious perception, with the support of cortical network states.^[^ ^177^ ^]^ In addition, diverse mental disorders are associated with the claustrum. In depression, bipolar disorder, and schizophrenia, the volume of the claustrum is reduced in patients, disease‐related genes are expressed, and the activity of each disease is altered.^[^ ^175^, ^178^ ^]^ Therefore, investigations of the interaction between serotonergic psychedelics and 5‐HT2AR in mental disorders are needed. For example, the administration of DOI at a dose of 6.0 mg kg^−1^ to rats resulted in a significant increase in c‐Fos levels in the neurons of the claustrum. c‐Fos is a marker of neuronal electrical activity and a transcription factor that responds to external stimuli, which is suggested to be enhanced by 5‐HT2AR activation.^[^ ^172^, ^179^ ^]^ In 2023, psilocybin at a dose of 1 mg kg^−1^ and ketamine at a dose of 10 mg kg^−1^ in mice elevated c‐Fos expression in various brain regions involving the claustrum, which might be associated with psychedelic‐induced increases in several glutamate receptor genes, such as *Grin2a* and *Grin2b*, as well as the 5‐HT2AR coding gene *Htr2a*.^[^ ^180^ ^]^ In 2024, the claustrum also revealed enrichment of 5‐HT2CRs, especially those expressed on glutamatergic neurons, and the neurons of this region project to the anterior cingulate cortex, which modulates EPSCs as postsynaptic responses through the 5‐HT2CR after the administration of DOI.^[^ ^181^ ^]^ However, several regions of the brain and 5‐HT2AR occupancy are restricted among the various receptors, which is insufficient evidence to define the accurate relationships and mechanisms between psychedelics and diverse receptors in the brain in individuals with mental disorders. Therefore, further studies are needed to obtain a comprehensive understanding of the pharmacological effects of spatial and cell types in the brain.

Spatial transcriptomics can provide critical information on how distinct components of various cell types state unique responses from each brain region and how individual cell types correlate with each other to promote specific functions through sequencing‐ and imaging‐based analyses from in situ hybridization (ISH) and in situ sequencing (ISS)‐based approaches.^[^ ^182^ ^]^ Spatial transcriptomics in neuroscience has been applied to resolve neural circuits, cell atlases, and brain activity, as well as to decipher the pathomechanisms between spatially pathogenic tissues and abnormally regulated molecular networks in brain disorders, such as neuropsychiatric and neurodegenerative disorders.^[^ ^169^, ^183^ ^]^ For example, the snRNA‐seq of the dorsolateral prefrontal cortex (DLPFC) from patients with MDD was distinguished into 26 cell‐type clusters using the identified 2135 genes across individual excitatory and inhibitory neurons, as well as nonneuronal cells—astrocytes, endothelial cells, oligodendrocytes, oligodendrocyte precursor cells (OPCs), and microglia. Compared with those in psychiatrically healthy individuals, abnormal gene expression was observed in almost half of the distracted cell types, particularly in OPCs and deep‐layer excitatory neurons. This altered expression of genes was associated with the dysregulation of synaptic plasticity from cytoskeletal modification, the fibroblast growth factor (FGF) pathway, the cycle of steroid hormone receptor (SHR), and immune function, which suggests the high consistency of differentially expressed genes (DEGs) compared with previous analyses in MDD.^[^ ^184^ ^]^ The spatial transcriptome overlaying snRNA‐seq data also revealed DEGs in the six layers of the DLPFC from patients with neuropsychiatric disorders, and the layer‐specific changes in gene expression were closely related to MDD, bipolar disorder, ASD, and schizophrenia. Layer 2 had enhanced gene expression overlapping the risk of ASD and bipolar disorders, and layer 5 presented enriched gene expression with a risk of schizophrenia.^[^ ^185^ ^]^ In 2024, spatial transcriptomic arrays were used to evaluate genome‐wide claustrum gene expression in sections from the claustrum of conditional *Nurr1* gene‐producing mice in which the *Nurr1* gene is known to be a transcription factor that is highly expressed in the claustrum. Through the analysis of spatial transcriptomic gene expression data, separate clusters were identified from WT and *Nurr1*‐deleted mice, which clearly occupied different spaces of the claustrum in spatial transcriptomic uniform manifold approximation and projection (UMAP). Furthermore, *Nurr1* deletion in the claustrum resulted in various DEGs, especially the downregulation of *bdnf*, *oprk1* (encoded κ opioid receptor) *5‐HT2AR*, and *5‐HT2CR*, which were identified by ISH as κ opioid receptor, 5‐HT2AR, and 5‐HT2CR, which are hallucinogen‐associated receptors that are abundant in the claustrum. These results suggest the potential for altered cortical functional connectivity from the claustrum, which may be related to differences in the efficiency of psychedelics.^[^ ^186^ ^]^ This evidence reinforces the importance of investigating the uniquely changed transcript profile from specific cell types localizing to distinct brain regions, which may lead to the identification of new targets and avenues for drug discovery within psychedelic drugs and mental disorders.

### 3.4 Emerging Approaches to Artificial Intelligence (AI)

**Figure 4:**
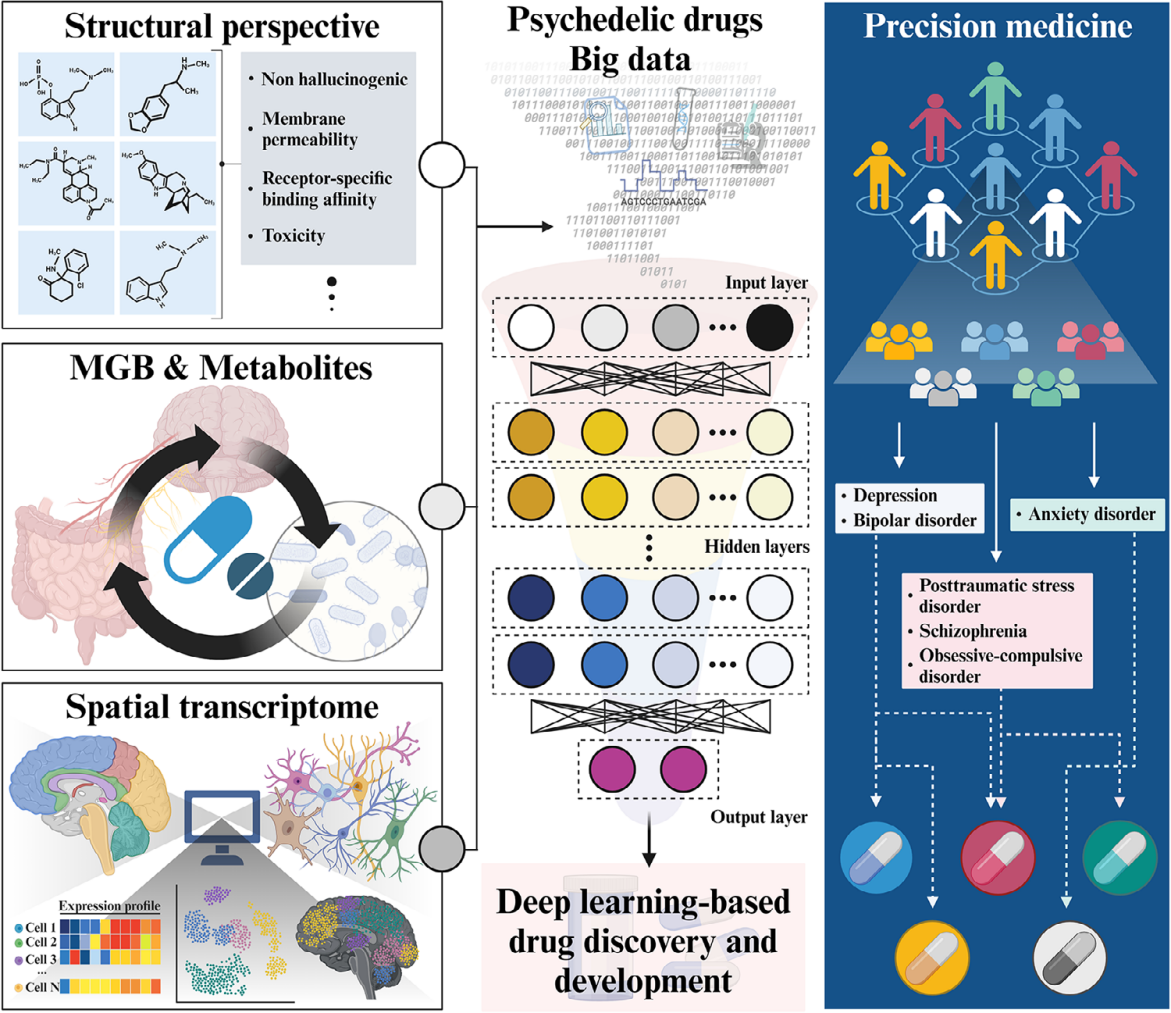
Deep learning‐based drug discovery and development in psychedelic drugs. The unintegrated mechanisms and different efficiencies of psychedelic drugs in mental disorders hinder the application of therapeutic drugs. These limitations have led to investigations of more multifaceted aspects of structural function, the microbiota‒gut‒brain (MGB) axis, and transcriptomes from specific cell types or brain regions, which may provide a comprehensive understanding of the psychopharmacology of psychedelic drugs to overcome the challenges associated with mental disorders. Through the accumulation of data on psychedelic drugs, deep learning‐based drug discovery and development can be used to predict the optimization of psychedelic drugs with biological mechanisms and interindividual genetics that affect the effects of pharmacological therapeutics on pharmacokinetics and pharmacodynamics. Artificial intelligence (AI)‐based models should be designed multiobjectively to respond to the multiple variables in drug discovery and development that may lead to advanced precision medicine from the individual mental disorders of patients. The figure was created with BioRender.com.

The transcriptome in the brain space is limited to only nonhuman models, so AI is anticipated to help find answers. To date, generative AI is not enough to be an all‐rounder; it has been kept within a particular usage range to help medical decisions: generalist medical AI (GMAI), BiomedGPT, and PathChat.^[^ ^187^ ^]^ However, the FDA has addressed AI as a critical player in the drug industry,^[^ ^188^ ^]^ and numerous methods have been suggested to adjust AI, such as predicting drug toxicity.^[^ ^189^ ^]^ Owing to revolutionizing the AI healthcare field, it also leads to mental disorders^[^ ^190^ ^]^ and is expected to have substantial promise in explosively growing areas.^[^ ^191^ ^]^ Although the prediction should be proven through experimental analysis of validity and accuracy via animal, health, or patient individuals, the ability of AI to prevent mental disorders seems not too far apart, as does psychedelic drug discovery (**Figure** **4**).

#### 3.4.1 Application in Mental Disorders

Several studies have attempted to develop novel approaches for pharmacogenomics or pharmacological targets in psychiatric disorders using ML^[^ ^192^ ^]^ or DL^[^ ^193^ ^]^ to integrate multiomics and neuroimaging data.^[^ ^194^ ^]^ For example, the ARPNet and ensemble learning models demonstrate the combined utilization of both pharmacogenomics and neuroimaging as multiomics data with single nucleotide polymorphisms (SNPs) and MRI data that can predict the response to treatment with early‐stage antidepressants in patients with MDD, with accuracies of approximately 84% and 86%, respectively. ARPNet can predict advanced prescriptions via inference and analysis of the similarity of patient features from data and previous treatment records of antidepressant drugs.^[^ ^195^ ^]^ Ensemble learning can also predict the drug response of MDD patients via six brain regions identified via fMRI, such as the right hippocampus, right posterior cingulate gyrus, right amygdala, left hippocampus, left anterior cingulate, left orbital part superior frontal gyrus, and paracingulate gyri, as well as the main SNPs involving *HTR2C* (rs1801412), *TOR1A* (rs3842225), and *DRD5* (rs1967550), which are associated with enhanced effects of SSRIs with the release of norepinephrine and dopamine.^[^ ^196^ ^]^ In 2024, deep learning‐based DiffMDD was suggested for the prediction of diagnostic tools by training EEG signals as a noninvasive technique using spontaneous electrical activity recordings.^[^ ^197^ ^]^

#### 3.4.2 Perspective of Drug Design and Discovery

ML and DL using big data involving genomics, proteomics, and clinical data have been suggested to overcome the complex challenges of drug discovery with respect to biological activity, off‐target effects, absorption, distribution, metabolism, excretion, and toxicity (ADMET), which can be used to predict and guide multiple aspects of drug design and discovery processes.^[^ ^198^ ^]^ Diverse AI‐based models involving ML and DL have been developed to predict the optimized procedures of drug discovery and development; DTI‐CNN, DeepCPI, DeepDTA, WideDTA, PADME, DeepAffinity, Deep docking, DeepBAR, and DeepFusion for drug‒target interaction and binding affinity; Alphafold and CASP for protein structure; ReLeaSE, DeepScaffold, AIScaffold, and DESMILES for de novo compound design; DeepVS, SIEVE score, and similarity search for virtual screening‐based hit identification; Metabolite prediction, Oral bioavailability prediction, and OpenChem for pharmacokinetic property; MultiCon for drug activity; Chemi‐Net for ADME property; MT‐DTI for drug‒protein interaction; DeepDTnet for drug target identification and repurposing; DeepCE for interaction between drugs and genes.^[^ ^199^ ^]^ In addition, drug‐induced side effects and toxicity are common causes that hinder the progress of candidate drugs and increase the risk and cost of drug development. AI‐based approaches have been predicted by several deep learning models.^[^ ^200^ ^]^ These include SDPred,^[^ ^201^ ^]^ DeepSide,^[^ ^202^ ^]^ and DruGNN^[^ ^199g^ ^]^ for drug side effects; DeepTox for diverse toxicity types^[^ ^198a^ ^]^; Deep‐PK^[^ ^203^ ^]^ and ToxMPNN^[^ ^204^ ^]^ for small‐molecule pharmacokinetics and toxicity; Tox_(R)CNN^[^ ^205^ ^]^ and DTox^[^ ^206^ ^]^ for drug cytotoxicity; DeepAOT for the oral toxicity of compounds;^[^ ^207^ ^]^ deephERG for human ether‐à‐go‐go‐related gene (hERG)‐mediated drug cardiotoxicity;^[^ ^208^ ^]^ PhenoTox for drug‐mediated structural toxicity in cardiomyocytes and hepatocytes;^[^ ^209^ ^]^ the consensus model for drug‐induced ototoxicity;^[^ ^210^ ^]^ the E3 model;^[^ ^211^ ^]^ and DILI deep learning^[^ ^212^ ^]^ for drug‐induced liver injury. There are several examples of successful evidence of drug discovery via DL.^[^ ^199^, ^213^ ^]^ DeepDTnet was used to predict the identification of targets and repurposed drugs via a drug‒gene‒disease network. Topotecan, an approved topoisomerase inhibitor, is a novel inhibitor of retinoic acid receptor‐related orphan receptor‐gamma t (ROR‐γt) that has a potent therapeutic effect on multiple sclerosis in mice.^[^ ^199b^ ^]^ DeepCE predicted that 10 drugs could be repurposed for COVID‐19 treatment through the prediction of the relationships between drugs and genes. In particular, three drugs, alisporivir, Cyclosporine, and Voclosporin, have critical functions in the life cycle of coronaviruses as cyclophilin enzyme inhibitors. This evidence has led to the need for clinical trials to determine their therapeutic efficacy.^[^ ^214^ ^]^ Similarly, MT‐DTI can predict the interaction between drugs and their target proteins, which is applied to determine the ability of atazanavir, an FDA‐approved therapeutic drug for HIV that interacts with the protein of SARS‐CoV‐2, to prevent COVID‐19.^[^ ^215^ ^]^ Indeed, AI‐based biocompanies have provided positive evidence.^[^ ^199^, ^213^ ^]^ Benevolent AI suggested the use of ruxolitinib and baricitinib for the treatment of COVID‐19 and BEN2293 for the treatment of atopic dermatitis. Among these drugs, baricitinib, a Janus kinase (JAK)1/JAK2 inhibitor approved for the treatment of rheumatoid arthritis, was predicted to be repurposed for the treatment of COVID‐19 through its anticytokine effects and suppression of viral propagation and received FDA approval to treat hospitalized COVID‐19 patients with baricitinib combined with remdesivir.^[^ ^216^ ^]^ AbCellera Biologics Inc. predicted and developed the COVID‐19 antibody LY‐CoV1404, which targets several SARS‐CoV‐2 variants, including B.1.1.7, B.1.351, P.1, B.1.526, B.1.428, B.1.429, and B.617.^[^ ^217^ ^]^ In other diseases, Exscientia designed drug candidates involving the adenosine receptor A2 (A2AR)/CD73 bispecific inhibitory molecules EXS21546 for the treatment of solid tumors and DSP‐1181 and 0038 as 5‐HT1A agonists and 5‐HT2A antagonists for the treatment of obsessive‒compulsive disorder and Alzheimer's disease psychosis.^[^ ^218^ ^]^ These approaches significantly reduce the consumption of time for drug development from initial screening to preclinical trial testing, which requires an average of approximately 4–6 years.

#### 3.4.3 Applicability of Psychedelic Drugs

The iSeroSnFR was developed as a fluorescent serotonin sensor with the redesign of a rapid binding pocket through machine learning to identify the circulation of serotonin in the millisecond range in freely moving mice during analysis of behavior tests that can be conjugated to the broad screening of changed serotonin between experimental groups and doses of SSRIs or serotonergic psychedelic drugs.^[^ ^219^ ^]^ This tool was based on the development of other fluorescent biosensors for serotonin, such as psychLight and sDarken, which are designed for each specific 5‐HT2A‐ and 5‐HT1AR‐based sensor and were used in the drug discovery of psychedelic analogs.^[^ ^148^, ^220^ ^]^ In 2024, Deep CANALs were developed to improve the deployment of practical conceptual frameworks to infer drug activity through the application of RElaxed Beliefs Under pSychedelics (REBUS) for psychedelics and CANAL for psychopathology.^[^ ^221^ ^]^ Briefly, the REBUS model indicates that psychedelics lead to modifications in the neurobiologically high level of beliefs encoded in the brain and explain the acute and psychedelic effects for therapeutic purposes,^[^ ^222^ ^]^ whereas the CANAL model simply canals behavioral and cognitive phenotypes such that the psychopathological component controls the psychological condition of individuals as the result of a response, including adversity, dysphoria, and distress.^[^ ^223^ ^]^ Although there is insufficient evidence to establish prediction tools for drug discovery between psychedelic drugs and mental disorders, this model may show the potential of an advanced paradigm that provides novel predictions to optimize psychedelic drugs from multiple aspects of psychopathology and connect to the most beneficial drugs for individuals.^[^ ^221^ ^]^ Therefore, the multifaceted range of analysis and integration using AI involving ML and DL is necessary to understand the mechanisms that lead to circumstantial changes in the brain to realize psychedelic therapy and how to design and develop psychedelic drugs.

## 4 Conclusion

Psychedelic drugs have been in the “gold rush” stage, and various studies and healthcare industries are focusing on achieving therapeutic applicability.^[^ ^134^, ^224^ ^]^ This paradigm has improved the understanding of the pharmacological mechanisms of psychedelic drugs through various receptors, but the unpredicted results have raised complicated challenges that have hindered the approval of drugs for the treatment of mental disorders. The abuse, long‐term effects, susceptibility of pregnant women, and harmful effects on newborns are continually increasing social and therapeutic challenges. As a result of education about hallucinogens, U.S. young‐aged groups have decreased in abusing several psychedelics from the past to 2022. Adult groups have shown increasing exposure to psychedelic drugs in the past 4 years, with a peak recorded in 2023, indicating the potential threat of the abuse of psychedelics.^[^ ^225^ ^]^ Individuals who experienced abnormal hallucinations within 72 hours after taking psychedelics reported their ongoing use of these substances, along with side effects such as negative thoughts and anxiety induction.^[^ ^226^ ^]^ Regarding the long‐term effects of psychedelic drugs, follow‐up studies after drug administration have varied in duration, with some studies lasting as short as 6 months, whereas more recent studies have extended periods of 1 to 3 years to assess the consequences of psychedelics for a long period of time.^[^ ^133a^ ^]^ A study with cancer patients revealed that approximately 20% of patients experienced recurrence of depression and anxiety during a 6‐month follow‐up.^[^ ^72^ ^]^ While psychedelic drugs can confer positive effects depending on the individual's condition, negative effects also occur, highlighting the need for further research and improvements in this area. The administration of psychedelic drugs during pregnancy is vulnerable to both the mother and the unborn child, which contributes to premature birth with an exceedingly low survival rate or damage and hormonal abnormality‐mediated infertility. Even exposure can cause teratogenicity, defective neurodevelopment, and toxicity in newborns.^[^ ^227^ ^]^ The eligible 96 pregnant women were administered MDMA and grouped by usage: a heavy group consumed an average of 3.3 (±4) tablets of MDMA before pregnancy, a light group consumed an average of 0.12 (±0.2) tablets, and a non‐MDMA group. The babies’ motor behavior decreased with MDMA exposure,^[^ ^228^ ^]^ and additional follow‐up investigations revealed motor delays between the ages of 4 months and 2 years.^[^ ^229^ ^]^ Although attention to the relationship between the susceptibility of pregnant women to psychiatric symptoms must be measured, ethical concerns may arise with psychedelics and pregnancy research to perform a larger scale. Therefore, it is essential to shift the study frame by conducting additional studies before advancing to further research in human beings.

Currently, the FDA has emphasized the importance of pharmacogenomics in many academic fields of disease to approve drugs for safety and efficiency for their intended purposes.^[^ ^230^ ^]^ Advanced genome analysis technologies have also reported correlations between pathological progress and the transcriptome, which continually highlights the importance of understanding the genome for drug discovery and therapeutics.^[^ ^231^ ^]^ Strategic approaches involving specific receptors, analogs, microdoses, and transcriptomics have been investigated to understand the pharmacological interactions between psychedelics and the brain, as well as their different effects on individuals. However, several questions remain concerning the importance of accessing psychedelic drugs, including the lack of significant evidence to demonstrate the direct correlation among key receptors, MGB, specific brain regions, and cell types over psychedelic treatments, as well as the distinct applicability between healthy individuals and patients with mental disorders.^[^ ^176^, ^232^ ^]^ ML and DL have been recommended as burgeoning tools that combine the individual data of patient‐associated features and medical records and learn to predict precisely through multiple input datasets. Recently, various ML and DL models have been used to predict mental disorders, drug response, and prescription, as well as successful cases in drug discovery and development. These AI‐based approaches predominantly decrease the consumption of time and cost during drug development from initial screening to preclinical trial testing and have the potential to overcome the complicated psychedelics effects of the multiple etiologies of mental disorders and interindividual differences. However, limitations remain in that they completely rely on the context of the input or trained dataset. Therefore, the training quality and quantity of the dataset are important because the results are directly correlated with the accuracy and precision of the analysis of the ML and DL methods.^[^ ^233^ ^]^ Additionally, drug discovery and development involve manifold processes such that a systematic multiobjective AI model is essential for responding to multiple variables.^[^ ^234^ ^]^ Likewise, further studies should be conducted from multiple perspectives to determine what directly influences individuals with genetic interplay and pharmacogenetics, where the main targeted regions of the brain or cell types are involved, and how specific cascades of psychiatric effects are engaged in pharmacokinetics and dynamics. As AI‐assisted approaches for drug discovery and development have advanced, predicting the psychedelics‐dependent impact on individual genetic changes and biological regulation is anticipated to maximize the therapeutic potency of psychedelic drugs and optimize precision medication for diverse patients with mental disorders.

## Conflict of Interest

The authors declare no conflict of interest.

## Author Contributions

S.‐H. K. and S. Y. contributed equally to this work. S.‐H.K. and S.Y. wrote the article. J.J. and J.C. contributed the researched data for the review. M.K. contributed substantially to conceptualization of the content in the article. All authors contributed substantially to discussion of the concept and edited the manuscript before submission. J.‐Y.J. supervised the project.
